# Discrete and Semi-Discrete Multidimensional Solitons and Vortices: Established Results and Novel Findings

**DOI:** 10.3390/e26020137

**Published:** 2024-02-02

**Authors:** Boris A. Malomed

**Affiliations:** Instituto de Alta Investigación, Universidad de Tarapacá, Casilla 7D, Arica 1000000, Chile; malomed@tauex.tau.ac.il

**Keywords:** nonlinear optics, Bose–Einstein condensates, lattice dynamics, nonlinear Schrödinger equations, vortices, skyrmions, Salerno model, spin–orbit coupling, quantum droplets, 𝒫𝒯 symmetry

## Abstract

This article presents a concise survey of basic discrete and semi-discrete nonlinear models, which produce two- and three-dimensional (2D and 3D) solitons, and a summary of the main theoretical and experimental results obtained for such solitons. The models are based on the discrete nonlinear Schrödinger (DNLS) equations and their generalizations, such as a system of discrete Gross–Pitaevskii (GP) equations with the Lee–Huang–Yang corrections, the 2D Salerno model (SM), DNLS equations with long-range dipole–dipole and quadrupole–quadrupole interactions, a system of coupled discrete equations for the second-harmonic generation with the quadratic ($\chi^{\left(2\right)}$) nonlinearity, a 2D DNLS equation with a superlattice modulation opening mini-gaps, a discretized NLS equation with rotation, a DNLS coupler and its PT-symmetric version, a system of DNLS equations for the spin–orbit-coupled (SOC) binary Bose–Einstein condensate, and others. The article presents a review of the basic species of multidimensional discrete modes, including fundamental (zero-vorticity) and vortex solitons, their bound states, gap solitons populating mini-gaps, symmetric and asymmetric solitons in the conservative and PT-symmetric couplers, cuspons in the 2D SM, discrete SOC solitons of the semi-vortex and mixed-mode types, 3D discrete skyrmions, and some others.

## 1. Introduction

### 1.1. Discrete Nonlinear Schrödinger Equations

#### 1.1.1. The Basic Equation

Commonly adopted models of physical media are based on linear and nonlinear partial differential equations, such as the Gross–Pitaevskii (GP) equations for the mean-field wave function $\psi \left(x,y,z;t\right)$ in atomic Bose–Einstein condensates (BECs) [1], and nonlinear Schrödinger (NLS) equations for the amplitude of the electromagnetic field in optical waveguides [2,3]. The scaled form of the three-dimensional (3D) GP/NLS equation is

(1) $$i\psi_{t}=-\left(1/2\right)\nabla^{2}\psi +\sigma {\left|\psi \right|}^{2}\psi +U\left(x,y,z\right)\psi ,$$

where σ=+1 and −1 correspond to the self-defocusing and focusing signs of the cubic nonlinearity and $U\left(x,y,z\right)$ is an external potential. In the application to optics, time *t* is replaced, as the evolution variable, by the propagation distance *z*, while the original coordinate *z* is then replaced by the temporal one, $\tau =t-z/V_{\mathrm{gr}}$, where $V_{\mathrm{gr}}$ is the group velocity of the carrier wave [2]. In optics, the effective potential may be two-dimensional (2D), with −U(x,y) representing a local variation of the refractive index in the transverse plane.

In many cases, the potential is spatially periodic, such as the one induced by optical lattices (OLs) in BECs [4,5,6], or by photonic crystals, which steer the propagation of light in optics [7]:

(2) $$U_{\mathrm{latt}}\left(x,y,z\right)=-\varepsilon \left[\cos(2\pi L/x)+\cos(2\pi L/y)+\cos(2\pi L/z)\right],$$

as well as its 2D and 1D reductions. A deep lattice potential, which corresponds to a large-amplitude ε in Equation (2), splits the continuous wave function into a set of “droplets” trapped in local potential wells, which are linearly coupled by tunneling. Accordingly, in the framework of the *tight-binding approximation,* the NLS equation is replaced by a discrete NLS (DNLS) equation with the linear coupling between adjacent sites of the discrete lattice (nearest neighbors). Such equations, with the second-order spatial derivatives replaced by their finite-difference counterparts, were derived (in the 1D form) for arrays of optical fibers [8,9,10,11] and plasmonic nanowires [12], as well as for BECs loaded in a deep OL potential [13]. The full 3D form of the DNLS equation is

(3) $$\begin{array}{c} i{\dot{\psi }}_{l,m,n}=-\left(1/2\right)\left[\left(\psi_{l+1,m,n}+\psi_{l-1,m,n}-2\psi_{l,m,n}\right)+\left(\psi_{l,m+1,n}+\psi_{l,m-1,n}-2\psi_{l,m,n}\right)\right.\\ \left.+\left(\psi_{l,m,n+1}+\psi_{l,m,n-1}-2\psi_{l,m,n}\right)\right]+\sigma {\left|\psi_{l,m,n}\right|}^{2}\psi_{l,m,n}+V_{l,m,n}\psi_{l,m,n}, \end{array}$$

where the overdot stands for d/dt and the set of integer indices, $\left(l,m,n\right)$, replaces the continuous coordinates $\left(x,y,z\right)$ in Equation (1). In Equation (3), the potential $V_{l,m,n}$ is a possible smooth addition to the deep lattice potential which imposes the discretization.

The rigorous mathematical derivation of the DNLS equation by the discretization of the underlying continuum NLS equation with the deep spatially periodic potential is based on the expansion of the continuous wave field over the set of Wannier functions [14]. These are linear combinations of the quasiperiodic Bloch wave functions, which feature shapes localized around potential minima [15], thus offering a natural basis for the transition to the discrete limit.

The full DNLS Equation (3) is often reduced to its 2D and 1D forms. The 1D lattices are sometimes built in the form of zigzag chains, making it relevant to add couplings between the next-nearest neighbors [16,17]. The 2D lattices with similar additional couplings were elaborated as well [18].

Unlike the continuous NLS Equation (1), the nonlinearity signs σ=±1 in Equation (3) are actually equivalent to each other. Indeed, the *staggering transformation* of the discrete wave function:

(4) $$\psi_{l,m,n}\left(t\right)\equiv {\left(-1\right)}^{l+m+n}\exp\left(-6it\right){\tilde{\psi }}_{l,m,n}^{*}\left(t\right),$$

where * stands for the complex conjugate, transforms Equation (3) with σ=−1 into the same equation for ${\tilde{\psi }}_{l,m,n}$ with σ=+1. For the 2D and 1D DNLS equations, $\exp\left(-6it\right)$ in the corresponding versions of staggering transformation (4) is replaced, respectively, by $\exp\left(-4it\right)$ and $\exp\left(-2it\right)$.

#### 1.1.2. Extended Equations

The DNLS equation and its extensions, such as systems of coupled DNLS equations [19,20], constitute a class of models with a large number of physical realizations [11,21,22,23,24]. They have also drawn much interest as subjects of mathematical studies [25]. One of the incentives for this interest is the fact that the discreteness arrests the development of the *critical* and *supercritical collapse*, which is driven by the self-focusing nonlinear term with σ=−1 in the 2D and 3D continuous NLS equations (1), respectively. The collapse leads to the emergence of singular solutions in the form of infinitely tall peaks, after a finite evolution time [3]. Naturally, the discreteness causes the arrest of the collapse, replacing it by a *quasi-collapse* [26], when the width of the shrinking peak becomes comparable to the spacing of the DNLS lattice.

The possibility of the collapse destabilizes formal 2D and 3D soliton solutions, which are produced by Equation (1); therefore, a challenging problem is the prediction and experimental realization of physical settings that make it possible to produce stable multidimensional solitons [27,28,29]. Thus, the discreteness provides a general method for the stabilization of 2D and 3D solitons.

##### The Gross–Pitaevskii Equations Amended by Effects of Quantum Fluctuations

Another promising possibility for the suppression of the collapse is offered by binary BECs, in which the cubic inter-component attraction creates 3D soliton-like states in the form of “quantum droplets” (QDs), while the development of the supercritical collapse is arrested by the self-repulsive quartic term, which takes into account a correction to the mean-field nonlinearity produced by quantum fluctuations (known as the celebrated *Lee–Huang–Yang* effect [30]). For the symmetric binary condensate, with identical wave functions ψ of its components, the accordingly amended scaled GP equations (in the absence of the trapping potential) was derived by Petrov [31]:

(5) $$i\psi_{t}=-\left(1/2\right)\nabla^{2}\psi -{\left|\psi \right|}^{2}\psi +{\left|\psi \right|}^{3}\psi .$$

Surprisingly quickly, the QD modes predicted by Equation (5) have been created experimentally, in the quasi-2D [32,33] and fully 3D [34] forms. The reduction of the spatial dimension to 2D and 1D replaces Equation (5) by the following (rescaled) GP equations, respectively [35]:

(6) $$\begin{array}{ccc} 2D:\;\; & & i\psi_{t}=-\left(1/2\right)\nabla^{2}\psi +{\left|\psi \right|}^{2}{\ln(|\psi |}^{2})\cdot \psi , \end{array}$$

(7) $$\begin{array}{ccc} 1D:\;\; & & i\psi_{t}=-\left(1/2\right)\psi_{xx}+{\left|\psi \right|}^{2}\psi -\left|\psi \right|\psi . \end{array}$$

Note, in particular, that, in the 1D Equation (7), the quantum correction is represented by the *attractive* term −|ψ|ψ, contrary to its repulsive counterpart $+{\left|\psi \right|}^{3}\psi$ in the 3D Equation (5). For this reason, the usual mean-field cubic term is taken in Equation (7) with the self-repulsion sign, to make it possible to study the effects of the competition of the quadratic self-attraction and cubic repulsion [36]. A semi-discrete version of Equation (7) is considered below, see Equation (66).

##### The Ablowitz–Ladik and Salerno–Model Equations

The 1D continuous NLS equation without the external potential is integrable by means of the inverse-scattering transform, with either sign of the nonlinearity, σ=±1 [37,38,39,40], although 2D and 3D extensions of the NLS equation are nonintegrable. The straightforward discretization destroys the integrability of the 1D NLS equation [41,42]. Nevertheless, the NLS equation admits a specially designed 1D discretization, which leads to an integrable discrete model, viz. the Ablowitz–Ladik (AL) equation [43]:

(8) $$i{\dot{\psi }}_{n}=-\left(\psi_{n+1}+\psi_{n-1}\right)\left(1+\mu {\left|\psi_{n}\right|}^{2}\right),$$

where the positive and negative values of the nonlinearity coefficient, μ, correspond to the self-focusing and defocusing, respectively. Integrable discrete equations, such as the AL one, are exceptional models, which provide exact solutions for discrete solitons [44].

Equation (8) gives rise to an exact solution for solitons in the case of μ>0. Setting μ≡+1 by means of rescaling, the solution is

(9) $$\psi_{n}\left(t\right)=\left(\sinh\beta \right)\mathrm{sech}\left[\beta (n-\xi (t))\right]\exp\left[i\alpha \left(n-\xi (t)\right)-i\varphi \left(t\right)\right],$$

where β and α are arbitrary real parameters that determine the soliton’s amplitude, A≡sinhβ, its velocity, $V\equiv \dot{\xi }=2\beta^{-1}\left(\sinh\beta \right)\sin\alpha$, and its overall frequency $\Omega \equiv \dot{\varphi }=-2\left[\left(\cosh\beta \right)\cos\alpha +\left(\alpha /\beta \right)\left(\sinh\beta \right)\sin\alpha \right]$. The existence of exact solutions for traveling solitons in the discrete system is a highly nontrivial property of the AL equation, which follows from its integrability. Generically, the motion of a discrete soliton through a lattice is braked by the emission of radiation, even if this effect may seem very weak in direct simulations [45]. Another integrable discrete model that admits exact solutions for moving solitons is the Toda-lattice equation for real coordinates $x_{n}\left(t\right)$ of particles with unit mass and the exponential potential of the interaction between adjacent ones [46]:

(10) $${\ddot{x}}_{n}+\;\exp\left(-\left(x_{n+1}-x_{n}\right)\right)-\exp\left(-\left(x_{n}-x_{n-1}\right)\right)=0.$$

Considerable interest has also been drawn to the nonintegrable combination of the AL and DNLS equations, in the form of the *Salerno model* (SM) [47], with an additional on-site cubic term, different from the intersite one in Equation (8):

(11) $$i{\dot{\psi }}_{n}=-\left(\psi_{n+1}+\psi_{n-1}\right)\left(1+\mu {\left|\psi_{n}\right|}^{2}\right)-2{\left|\psi_{n}\right|}^{2}\psi_{n}.$$

Here, the signs and magnitude of the on-site nonlinearity coefficient are fixed by means of the staggering transformation (4) and rescaling. The SM finds a physical realization in the context of the Bose–Hubbard model, which represents the BEC loaded in a deep OL, taking into account the nonlinearity of the intersite coupling [48].

The AL and SM Equations (8) and (11) conserve the total norm *N*, whose definition is different from the straightforward one for the DNLS equation, given below by Equation (27), namely

(12) $$N_{\mathrm{AL},\mathrm{SM}}=\left(1/\mu \right)\sum_{n}\ln\left|1+\mu |\psi_{n}{|}^{2}\right|,$$

see Refs. [43,49]. The Hamiltonians of the AL and SM equations, which are dynamical invariants as well, are

(13) $$H_{\mathrm{AL}}=-\sum_{n}\left(\psi_{n}\psi_{n+1}^{*}+\psi_{n+1}\psi_{n}^{*}\right),$$

(14) $$H_{\mathrm{SM}}=-\sum_{n}\left[\left(\psi_{n}\psi_{n+1}^{*}+\psi_{n+1}\psi_{n}^{*}\right)+\left(2/\mu \right){\left|\psi_{n}\right|}^{2}\right]+\left(2/\mu \right)N_{\mathrm{SM}}.$$

In particular, the ostensible “simplicity” of the Hamiltonian (13) is related to the complexity of the respective Poisson brackets (symplectic structure), which determine the evolution equations for $\psi_{n}$ as $d\psi_{n}/dt=\left\{H,\psi_{n}\right\}$. For the AL and SM models, the Poisson brackets, written for a pair of arbitrary functions $B\left(\psi_{n},\psi_{n}^{*}\right)$ and $C\left(\psi_{n},\psi_{n}^{*}\right)$, are [47,48]

(15) $$\left\{B,C\right\}=i\sum_{n}\left(\frac{\partial B}{\partial \psi_{n}}\frac{\partial C}{\partial \psi_{n}^{*}}-\frac{\partial B}{\partial \psi_{n}^{*}}\frac{\partial C}{\partial \psi_{n}}\right)\left(1+\mu {\left|\psi_{n}\right|}^{2}\right).$$

It is also relevant to consider the continuum limit of the SM, which is introduced by approximating the intersite combination of the discrete fields by a truncated Taylor’s expansion:

(16) $$\psi_{n}\left(t\right)\equiv e^{2it}\Psi \left(x,t\right),\;\Psi \left(x=n\pm 1,t\right)\approx \Psi \left(x=n\right)\pm \Psi_{x}{|}_{x=n}+\left(1/2\right)\Psi_{xx}{|}_{x=n}\;,$$

where Ψ(x) is considered as a function of continuous coordinate *x*, whose integer values coincide with the discrete lattice coordinate *n*. The substitution of the approximation (16) in Equation (11) leads to a generalized (nonintegrable) NLS equation [50]:

(17) $$i\Psi_{t}=-\left(1+\mu {\left|\Psi \right|}^{2}\right)\Psi_{xx}-2\left(1+\mu \right){\left|\Psi \right|}^{2}\Psi .$$

Equation (17) conserves the total norm and Hamiltonian, which are continuum counterparts of the expressions (12) and (14):

(18) $$\begin{array}{ccc} {\left(N_{\mathrm{AL}}\right)}_{\mathrm{cont}} & = & \frac{1}{\mu }\int_{-\infty }^{+\infty }dx\;\ln\left|1+{\mu |\Psi |}^{2}\right|, \end{array}$$

(19) $$\begin{array}{ccc} {\left(H_{\mathrm{SM}}\right)}_{\mathrm{cont}} & = & \int_{-\infty }^{+\infty }dx\left[{\left|\Psi_{x}\right|}^{2}-2\left(\frac{1}{\mu }+1\right){\left|\Psi \right|}^{2}\right]+\frac{2}{\mu }{\left(N_{\mathrm{AL}}\right)}_{\mathrm{cont}}\;. \end{array}$$

It is relevant to mention that the general approximation opposite to the continuum limit is the anti-continuum limit [21,51]. This approach starts with the limit form of the DNLS equation, in which the linear couplings between adjacent sites are dropped. Then, one can try to construct various states, including solitons, by introducing an input composed of simple solutions of the single-site equations corresponding to Equation (3), viz. $i{\dot{\psi }}_{l,m,n}=\sigma {\left|\psi_{l,m,n}\right|}^{2}\psi_{l,m,n}+V_{l,m,n}\psi_{l,m,n}$, at a finite set of sites, and keeping the zero solution at all others. The single-site “simple solutions” are

(20) $$\psi_{l,m,n}=a_{l,m,n}\exp\left(-i{\left|a_{l,m,n}\right|}^{2}t-iV_{l,m,n}t\right),$$

where $a_{l,m,n}$ is an arbitrary set of complex amplitudes. Next, one reintroduces weak linear intersite couplings and attempts to identify nontrivial solutions that may, thus, appear from the finite-set input composed of the single-site solutions (20).

##### Self-Trapping in Lattices with the Self-Repulsion Strength Growing from the Center to the Periphery

DNLS equations with the on-site self-repulsive nonlinearity, corresponding to σ>0 in Equation (3), may support discrete-soliton (self-trapped) states without resorting to the staggering transform (4) if the local self-repulsion strength is made a function of the lattice coordinates, growing fast enough from the center to the periphery. Originally, this option was elaborated in the framework of the continuum NLS and GP equations in the space of dimension *D*, with the local self-defocusing (repulsion) coefficients growing at r→∞ (*r* is the radial coordinate) faster than $r^{D}$ [52]. In terms of the 1D and 2D DNLS equations, similar settings were introduced in Refs. [53,54], with the site-dependent self-attraction coefficients, ${\left(\sigma_{n}\right)}_{1D}=\sigma_{0}\exp\left(\alpha |n|\right)$ and ${\left(\sigma_{m,n}\right)}_{2D}=\sigma_{0}\exp\left(\alpha \left(|m|+|n|\right)\right)$, respectively, with positive constants $\sigma_{0}$ and α. In the 2D model, solutions were constructed, and their stability analyzed, for fundamental, dipole, quadrupole, and vortical discrete solitons [52].

##### Discrete Nonlinear Schrödinger Equations with Long-Range Dipole–Dipole and Quadrupole–Quadrupole Intersite Interactions

It is well known that atomic BECs formed of ultracold atoms carrying permanent magnetic moments feature specific dynamical effects due to the long-range interactions between atomic moments [55,56]. This fact suggests combining the dipole–dipole interactions and a deep OL potential, thus introducing DNLS equations with the nonlocal (long-range) coupling between lattice sites. In the 2D setting, this model gives rise to different forms of the DNLS equations. The simplest setup corresponds to the case when the atomic moments are polarized by an external DC magnetic field perpendicular to the system’s plane. In this case, the dipole–dipole interactions amount to the isotropic nonlocal repulsion, accounted for by the respective interaction coefficient Γ>0 [57].

(21) $$\begin{array}{ccc} i\frac{\partial \psi_{m,n}}{\partial t} & = & -\frac{1}{2}\left(\psi_{m+1,n}+\psi_{m-1,n}+\psi_{m,n+1}+\psi_{m,n-1}-4\psi_{m,n}\right)+\left[\sigma {\left|\psi_{m,n}\right|}^{2}\right.\\ & & \left.+\Gamma \sum_{\left\{m^{\prime },n^{\prime }\right\}\neq \left\{m,n\right\}}{\left|{\left(m-m^{\prime }\right)}^{2}+{\left(n-n^{\prime }\right)}^{2}\right|}^{-3/2}{\left|\psi_{m^{\prime },n^{\prime }}\right|}^{2}\right]\psi_{m,n}\;, \end{array}$$

where σ is the same coefficient of the on-site self-interaction as in Equation (3). A more sophisticated setup corresponds to the atomic magnetic moments polarized parallel to the system’s plane. In the former case, the nonlocal term in the respective DNLS equation is anisotropic, being attractive in one in-plane direction and repulsive in the other, cf. Ref. [58]:

(22) $$\begin{array}{c} i\frac{\partial \psi_{m,n}}{\partial t}=-\frac{1}{2}\left(\psi_{m+1,n}+\psi_{m-1,n}+\psi_{m,n+1}+\psi_{m,n-1}-4\psi_{m,n}\right)+\left[\sigma {\left|\psi_{m,n}\right|}^{2}\right.\\ \left.+\Gamma \sum_{\left\{m^{\prime },n^{\prime }\right\}\neq \left\{m,n\right\}}\frac{{\left(n-n^{\prime }\right)}^{2}-2{\left(m-m^{\prime }\right)}^{2}}{{\left[{\left(m-m^{\prime }\right)}^{2}+{\left(n-n^{\prime }\right)}^{2}\right]}^{5/2}}{\left|\psi_{m^{\prime },n^{\prime }}\right|}^{2}\right]\psi_{m,n}\;. \end{array}$$

The analysis reported in Ref. [57] demonstrates that the nonlocal repulsion, added to Equation (21), helps to stabilize discrete solitons with embedded vorticity. Solutions of Equation (22) for anisotropic vortex solitons can be found as well, but they are completely unstable [57].

A 2D DNLS model that combines the local on-site nonlinearity and long-range interaction between particles carrying permanent quadrupole electric moments was elaborated in Ref. [59]:

(23) $$\begin{array}{c} i\frac{\partial \psi_{m,n}}{\partial t}=-\frac{1}{2}\left(\psi_{m+1,n}+\psi_{m-1,n}+\psi_{m,n+1}+\psi_{m,n-1}-4\psi_{mn}\right)+\left[\sigma |\psi_{mn}{|}^{2}\right.\\ \left.+\Gamma \sum_{\left\{m^{\prime },n^{\prime }\right\}\neq \left\{m,n\right\}}\frac{{\left(n-n^{\prime }\right)}^{2}-4{\left(m-m^{\prime }\right)}^{2}}{{\left[{\left(m-m^{\prime }\right)}^{2}+{\left(n-n^{\prime }\right)}^{2}\right]}^{7/2}}{\left|\psi_{m^{\prime }n^{\prime }}\right|}^{2}\right]\psi_{m,n}; \end{array}$$

cf. Equation (21). This model also gives rise to families of stable 2D discrete solitons [59].

##### The 2D Discrete Second-Harmonic-Generating ($\chi^{\left(2\right)}$) System

The quadratic (alias $\chi^{\left(2\right)}$) nonlinearity is a fundamentally important effect, which gives rise to coherent generation of the second harmonic in optics. In terms of the 2D spatial-domain propagation in a continuum material, the standard $\chi^{\left(2\right)}$ system for amplitudes $\psi \left(x,y,z\right)$ and $\phi \left(x,y,z\right)$ of the fundamental-frequency (FF) and second-harmonic (SH) waves is [60]

(24) $$\begin{array}{ccc} i\psi_{z} & = & -\left(1/2\right)\nabla^{2}\psi -\psi^{*}\phi \;,\\ 2i\phi_{z} & = & -\left(1/2\right)\nabla^{2}\phi -Q\phi -\frac{1}{2}\psi^{2}\;, \end{array}$$

where *z* is the propagation distance, the paraxial diffraction operator $\left(1/2\right)\nabla^{2}$ acts on the transverse coordinates $\left(x,y\right)$, *Q* is a real mismatch parameter, and * stands for the complex conjugate. The discretized version of Equation (24), which represents, in the tightly binding approximation, light propagation in a photonic crystal made of the $\chi^{\left(2\right)}$ material, is [61]

(25) $$\begin{array}{ccc} i\frac{d\psi_{m,n}}{dz} & = & -C_{1}\left(\psi_{m+1,n}+\psi_{m-1,n}+\psi_{m,n+1}+\psi_{m,n-1}-4\psi_{mn}\right)-\psi_{m,n}^{*}\phi_{m,n}\;,\\ 2i\frac{d\phi_{m,n}}{dz} & = & -\frac{C_{2}}{2}\left(\phi_{m+1,n}+\phi_{m-1,n}+\phi_{m,n+1}+\phi_{m,n-1}-4\phi_{mn}\right)-Q\phi_{m,n}-\frac{1}{2}\psi_{m,n}^{2}\;, \end{array}$$

where $C_{1}$ and $C_{2}$ are effective lattice-coupling constants for the FF and SH waves. The role of the conserved norm of the discrete $\chi^{\left(2\right)}$ system is played by the Manley–Rowe invariant, i.e., the total optical power, $I=\sum_{m,n}\left({\left|\psi_{m,n}\right|}^{2}+4{\left|\phi_{m,n}\right|}^{2}\right)$.

An essential property of 2D discrete solitons produced by Equation (25) is their mobility [61]. In this connection, it is relevant to mention that, while the development of the quasi-collapse in the 2D discrete NLS equation with the cubic self-attraction is arrested by the underlying lattice structure, the quasi-collapse strongly pins the 2D solitons to the same structure and, thus, makes them immobile. On the other hand, the $\chi^{\left(2\right)}$ nonlinearity does not give rise to the collapse in the 2D (and 3D) space; therefore, 2D $\chi^{\left(2\right)}$ solitons do not demonstrate a trend for strong pinning, remaining effectively mobile robust localized modes [61].

### 1.2. One-Dimensional DNLS Solitons

#### 1.2.1. Fundamental Solitons

In the 1D setting, the model of basic interest is the DNLS equation with self-attraction, which corresponds to σ=−1 in the 1D version of Equation (3), without the external potential ($V_{l,m,n}=0$):

(26) $$i{\dot{\psi }}_{n}=-\left(1/2\right)\left(\psi_{n+1}+\psi_{n-1}-2\psi_{n}\right)-{\left|\psi_{n}\right|}^{2}\psi_{n}\;.$$

This equation conserves two dynamical invariants, viz. the total norm:

(27) $$N=\sum_{n=-\infty }^{+\infty }{\left|\psi_{n}\right|}^{2},$$

and Hamiltonian (energy):

(28) $$H=\sum_{n=-\infty }^{+\infty }\left[\left(1/2\right){\left|\psi_{n}-\psi_{n-1}\right|}^{2}-\left(1/4\right){\left|\psi_{n}\right|}^{4}\right].$$

Stationary solutions to Equation (26) with real frequency ω are looked for as

(29) $$\psi_{n}\left(t\right)=e^{-i\omega t}u_{n},$$

with real amplitudes $u_{n}$ satisfying the discrete equation:

(30) $$\omega u_{n}=-\left(1/2\right)\left(u_{n+1}+u_{n-1}-2u_{n}\right)-u_{n}^{3}.$$

Note that Equation (30) can be derived by varying its Lagrangian,

(31) $$L=\sum_{n=-\infty }^{+\infty }\left\{\frac{1}{4}\left[{\left(u_{n}-u_{n-1}\right)}^{2}-u_{n}^{4}\right]-\frac{\omega }{2}u_{n}^{2}\right\},$$

with respect to the discrete real field $u_{n}$.

A fundamental property of the DNLS Equation (26) with the self-attractive on-site nonlinearity is the modulational instability (MI) of its spatially homogeneous continuous-wave (CW) state [62], $\psi_{n}=a\exp\left(ia^{2}t\right)$, with an arbitrary amplitude *a* (cf. Equation (29)). MI breaks the CW state into a chain of discrete solitons [11]. Analytical solutions for these solitons are not available, as the DNLS equation is not integrable. The solitons can be readily found in a numerical form and studied in the framework of the variational approximation (VA) [25]. The VA is based on a particular *ansatz*, i.e., an analytical expression, which aims to approximate the solution [63]. The only discrete ansatz for which analytical calculations are feasible is represented by the exponential function [64,65,66,67], namely

(32) $${\left(u_{n}\right)}_{\mathrm{on}-\mathrm{site}}=A\exp\left(-a|n|\right),$$

with a>0. The corresponding norm, calculated as per Equation (27), is

$$N_{\mathrm{ansatz}}=A^{2}\coth a.$$

**Figure 1:**
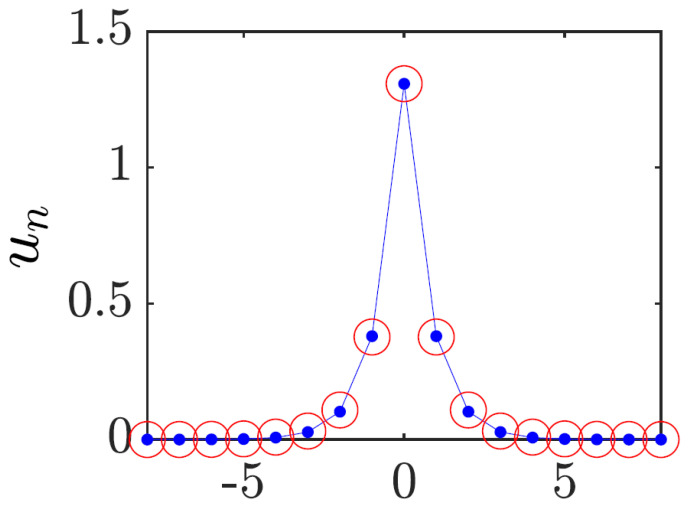
Comparison of a typical discrete soliton, obtained as the numerical solution of Equation (30), shown by chains of blue dots, and its counterpart produced by the VA (shown by red open circles). In this figure, ω=−1 (see Equation (29)), the corresponding parameters of the ansatz (32) being A≈1.31, a≈1.15. The figure is borrowed from Ref. [24].

Note that the ansatz (32) is appropriate for strongly and moderately discrete solitons, as shown in Figure 1, but not for broad (quasi-continuum) ones, which may be approximated by the commonly known soliton solution of the NLS equation (the 1D version of (1) with U=0):

(33) $$\psi \left(x,t\right)=\eta \;\mathrm{sech}\left(\eta \left(x-\xi \right)\right)\exp\left(i\eta^{2}t\right),$$

with width $\eta^{-1}$, which must be large in comparison with the discreteness spacing, $\eta^{-1}\gg 1$, and central coordinate ξ.

The substitution of the ansatz (32) in Lagrangian (31) produces the corresponding VA Lagrangian:

(34) $$L_{\mathrm{VA}}=\left(A^{2}/2\right)\tanh\left(a/2\right)-\left(A^{4}/4\right)\coth\left(2a\right)-\left(\omega /2\right)A^{2}\coth a.$$

Then, for given ω<0 (solitons do not exist for ω>0), the squared amplitude, $A^{2}$, and inverse width, *a*, of the discrete soliton are predicted by the Euler–Lagrange equations:

(35) $$\frac{\partial L_{\mathrm{eff}}}{\partial \left(A^{2}\right)}=\frac{\partial L_{\mathrm{eff}}}{\partial a}=0.$$

This corresponding system of algebraic equations for $A^{2}$ and *a* can be easily solved numerically. The VA produces accurate predictions for the solitons, as shown in Figure 1 and Ref. [68].

Rigorous justification of the VA was elaborated in Ref. [69]. Furthermore, the VA and a full numerical solution of Equation (26) demonstrate that the entire family of the discrete solitons is stable [25].

In addition to the bright solitons considered here, the DNLS equation also gives rise to discrete dark solitons, which have been studied in detail theoretically and experimentally [70,71,72,73]. As concerns the topic of the present review, two-dimensional discrete dark modes, such as delocalized lattice vortices, were studied as well [74]. However, detailed consideration of dark modes is not included in this article.

#### 1.2.2. Higher-Order One-Dimensional Modes: Twisted Discrete Solitons and Bound States

In addition to the fundamental (single-peak) solitons outlined above, Equation (30) admits stable second-order states in the form of *twisted modes*, which are subject to the antisymmetry condition, $u_{n}=-u_{1-n}$ [75]. Such states exist and are stable only in a strongly discrete form, vanishing in the continuum limit. In particular, the above-mentioned anti-continuum approximation is appropriate for the construction of the twisted modes.

Stable 1D DNLS solitons may form bound states, which also represent higher-order modes of the DNLS equation. They are stable in the *out-of-phase* form, i.e., with opposite signs of the constituent solitons [76,77], which resembles the structure of the twisted modes (however, the tight antisymmetric structure of the twisted modes cannot be considered as a bound state of fundamental solitons), the same being true for 2D DNLS solitons [78]. Note that stationary bound states of fundamental solitons do not exist in the continuum limit.

#### 1.2.3. One-Dimensional Solitons in the Salerno Model

The substitution of the usual ansatz (29) in Equation (11) produces a stationary discrete equation for real $u_{n}$:

(36) $$\omega u_{n}=-\left(u_{n+1}+u_{n-1}\right)\left(1+\mu u_{n}^{2}\right)-2u_{n}^{3};$$

cf. Equation (30). Discrete solitons produced by the SM Equation (11) with μ>0, i.e., with *noncompeting* intersite and on-site self-focusing nonlinearities, were investigated by means of numerical methods [49,79,80]. It was found that the SM gives rise to the 1D solitons at all positive values of μ.

Another option is to consider the SM with μ<0, which features *competing nonlinearities*, as the intersite cubic term, with the coefficient μ<0 in Equation (11), which accounts for the nonlinear coupling between adjacent sites of the lattice, and the on-site cubic term in Equation (11) represent, respectively, repulsive and attractive nonlinear interactions. This version of the SM gives rise to families of discrete solitons, in the usual form (29), with ω<0 and real $u_{n}$, of two different types. One family represents ordinary discrete solitons, similar to those generated by the DNLS equation. Another family represents *cuspons*, featuring a higher curvature of their profile at the center. A small subfamily of ordinary solitons produced by the SM with the competing nonlinearities is unstable, while all cuspons are stable.

As mentioned above, the antisymmetric bound states of DNLS solitons are stable, while symmetric bound states are unstable [76,77]. The same is true for bound states of ordinary discrete solitons in the SM [50]. However, in the framework of the SM with the competing nonlinearities, the situation is *exactly opposite* for the cuspons: their symmetric and antisymmetric bound states are stable and unstable, respectively [50].

### 1.3. The Subject and Structure of the Present Article

The above-mentioned reviews [11,21,22,23,24,25] produce a comprehensive survey of theoretical and experimental results for discrete solitons in various 1D systems. The objective of this article is to produce a relatively brief summary of results for multidimensional (chiefly, two-dimensional) discrete and semi-discrete solitons, which were considered in less detail in previous reviews and, on the other hand, draw growing interest in the context of the current work with 2D and 3D solitons in diverse physical contexts [27,28]. Furthermore, the presence of the two or three coordinates makes it also possible to define *semi-discrete* states as ones that are discrete in one direction and continuous in the perpendicular one [81,82,83,84].

This article chiefly represents theoretical results, but some experimental findings for quasi-discrete 2D solitons in photonic lattices [85,86] are included as well.

The review presented below does not claim to be comprehensive. It comprises results that are produced by conservative models of the DNLS types (including, in particular, the 2D SM). Discrete models of other types—in particular, those similar to the Toda lattice, see Equation (10), Fermi–Pasta–Ulam–Tsingou lattices [87], and Frenkel–Kontorova systems [88]—are not considered here. Dissipative systems are not considered either, except for a 2D model with the parity-time (PT) symmetry [89]; see Equation (70) below.

The rest of the article is arranged as follows. Basic results for fundamental (zero-vorticity) and vortex solitons, as well as bound states of such solitons, produced by the 2D DNLS equation and its generalizations, are summarized in Section 2, which is followed, in Section 3, by a brief consideration of fundamental and vortex solitons in the 2D Salerno model (SM). Section 4 addresses discrete solitons of the semi-vortex and mixed-mode types in the 2D spin–orbit-coupled (SOC) system of GP equations for a two-component BEC. Basic results for discrete self-trapped modes produced by 3D DNLS equations, including fundamental and vortex solitons, along with skyrmions, are presented in Section 5. The findings for 2D *semi-discrete* systems, again including fundamental and vortex solitons, supported by combined quadratic–cubic and cubic–quintic nonlinearities (which are relevant for BECs and optics, respectively), are summarized in Section 6. This section also addresses the transverse mobility of confined spatiotemporal modes in an array of optical fibers with the intrinsic cubic self-focusing (Kerr nonlinearity). Fundamental and vortex solitons produced by a PT-symmetric discrete 2D coupler with the cubic nonlinearity are considered in Section 7. The article is concluded in Section 8, which, in particular, suggests directions for the further work in this area and mentions particular topics that are not included in the present review.

## 2. Two-Dimensional Nonlinear Schrödinger Lattices: Fundamental and Vortex Solitons and Their Bound States

### 2.1. Vortex Solitons: Theoretical and Experimental Results

The basic 2D cubic DNLS equation is the 2D version of Equation (3) with the self-attraction (σ=−1) and without the external potential:

(37) $$i{\dot{\psi }}_{m,n}=-\left(1/2\right)\left(\psi_{m+1,n}+\psi_{m-1,n}+\psi_{m,n+1}+\psi_{m,n-1}-4\psi_{m,n}\right)-{\left|\psi_{m,n}\right|}^{2}\psi_{m,n};$$

cf. Equation (26). The substitution of $\psi_{m,n}=\exp\left(-i\omega t\right)u_{m,n}$ in Equation (37) produces the stationary equation, where the stationary discrete wave function, $u_{m,n}$, may be complex:

(38) $$\omega u_{m,n}=-\left(1/2\right)\left(u_{m+1,n}+u_{m-1,n}+u_{m,n+1}+u_{m,n-1}-4u_{m,n}\right)-{\left|u_{m,n}\right|}^{2}u_{m,n};$$

cf. (30). Fundamental soliton solutions to Equation (38) can be predicted by means of VA [90,91], using an exponential ansatz; see Equation (47) below (cf. Equation (32) for the 1D soliton). More interesting in the 2D setting are discrete solitons with *embedded vorticity*, which were introduced in Ref. [92] (see also Ref. [93]). Vorticity, alias the topological charge or winding number, is defined as $\Delta \varphi /\left(2\pi \right)$, where Δφ is a total change of the phase of the complex discrete function $u_{m,n}$ along a contour surrounding the vortex pivot. Stability is an important issue for 2D discrete solitons, because, in the continuum limit, the 2D NLS equation gives rise to the well-known Townes solitons [94], which are unstable against the onset of the critical collapse [3]. In the same limit, the Townes solitons with embedded vorticity (vortex rings [95]) are subject to much stronger instability against spontaneous splitting of the ring into fragments [96].

**Figure 2:**
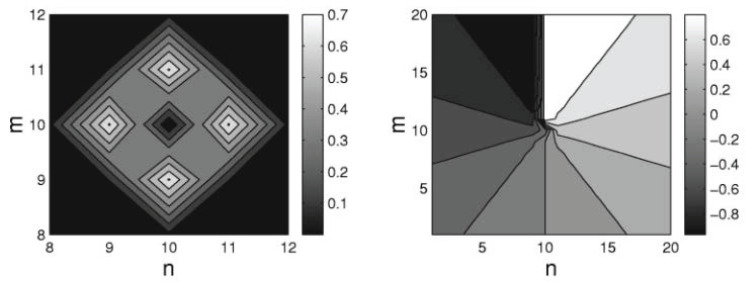
A stable discrete vortex soliton with topological charge S=1, produced by Equation (38) with ω=−3.2. The left and right panels show, respectively, the distributions of the absolute value and phase of the complex wave function $u_{m,n}$ in the plane with coordinates $\left(m,n\right)$. The figure is borrowed from Ref. [92].

The lattice structure of the DNLS equation provides for the stabilization of both fundamental (zero-vorticity) and vortex solitons [92]. A typical example of a stable 2D vortex soliton with topological charge S=1 is displayed in Figure 2. The 2D fundamental and vortex solitons, with topological charges S=0 and 1, are stable in the regions $-\omega >|\omega_{\mathrm{cr}}^{\left(S=0\right)}|\approx 0.50$ and $-\omega >|\omega_{\mathrm{cr}}^{\left(S=1\right)}|\approx 1.23$, respectively [92], while the higher-order discrete vortices with charges S=2 and 4 are unstable, being replaced by stable modes in the form of quadrupoles and octupoles [97]. The vortex solitons with S=3 may be stable, but only in an extremely discrete form, viz. at $-\omega >|\omega_{\mathrm{cr}}^{\left(S=2\right)}|\approx 4.94$. In agreement with what was said above, these results imply that all the solitons are unstable in the continuum limit, corresponding, in the present notation, to ω→0.

The experimentally relevant lattice structure may be anisotropic, with the linear combination $\left(\psi_{m+1,n}+\psi_{m-1,n}+\psi_{m,n+1}+\psi_{m,n-1}-4\psi_{m,n}\right)$ in Equation (37) replaced by $\left(\alpha \left(\psi_{m+1,n}+\psi_{m-1,n}\right)+\left(\psi_{m,n+1}+\psi_{m,n-1}\right)-2\left(1+\alpha \right)\psi_{m,n}\right),$ with the anisotropy parameter α≠1. Effects of the anisotropy on the structure and stability of the fundamental and vortical discrete solitons were explored in Ref. [98].

**Figure 3:**
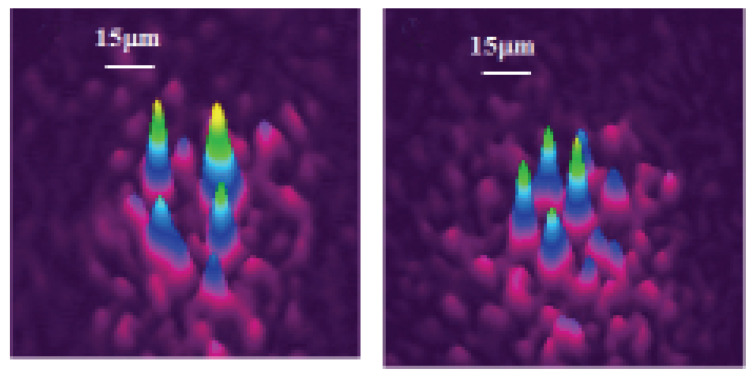
Quasi-discrete optical solitons with vorticity S=1, created in the photorefractive crystal with an induced deep photonic lattice. The left and right panels display, respectively, the on-site- and intersite-centered vortex solitons. The figure is borrowed from Ref. [86].

The theoretically predicted 2D discrete solitons with vorticity S=1 were experimentally created in Refs. [85,86], using a photorefractive crystal. Unlike uniform media of this type, where delocalized (“dark”) optical vortices were originally demonstrated [99,100], those works made use of a deep virtual photonic lattice as a quasi-discrete structure supporting the self-trapping of nonlinear modes in the optical field with extraordinary polarization (while the photonic lattice was induced as the interference pattern of quasi-linear beams in the ordinary polarization). Intensity distributions observed in vortex solitons of the on-site- and intersite-centered types (i.e., with the vortex pivot coinciding with a site of the underlying lattice, or set between the sites, respectively) are displayed in Figure 3.

Another interesting result demonstrated (and theoretically explained) in deep virtual photonic lattices is the possibility of periodic flipping of the topological charge of a vortex soliton initially created with the topological charge S=2 [101]. Stable vortex solitons with S=2 were created using a hexagonal virtual photonic lattice (while, as mentioned above, the localized modes with S=2 are completely unstable in the case of the square lattice) [102].

### 2.2. Bound States of 2D Discrete Solitons and Solitary Vortices

**Figure 4:**
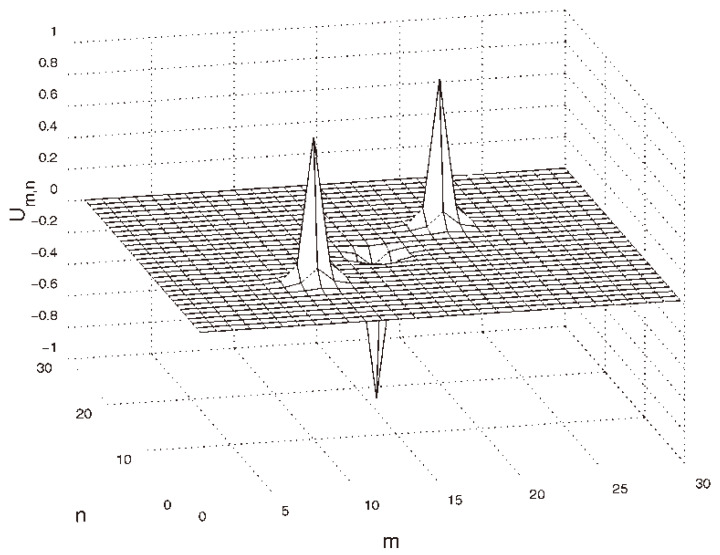
An example of a stable bound state of three 2D fundamental (zero-vorticity) solitons, which are mutually out of phase. The solution is produced by the numerical solution of Equation (38) with ω=−1. The figure is borrowed from Ref. [78].

As mentioned above, stable 2D discrete solitons may form stable bound states, composed of two or several items. Vortex solitons may form bound states as well. This possibility and stability of the resulting bound states are determined by an effective potential of interaction between two identical discrete solitons with intrinsic vorticity *S*, which are separated by a large distance *L*. The potential can be derived from an asymptotic expression for exponentially decaying tails of the soliton. In the quasi-continuum approximation, it is $u_{m,n}∼{\left(m^{2}+n^{2}\right)}^{-1/4}\exp\left(-\sqrt{-2\omega \left(m^{2}+n^{2}\right)}\right)$ (recall soliton solutions to Equation (38) exist for ω<0). Then, the overlap of the tail of each soliton with the central body of the other one gives rise to the following interaction potential:

(39) $$U_{\mathrm{int}}\left(L\right)\approx \mathrm{const}\cdot \cos\delta \cdot {\left(-1\right)}^{S}L^{-1/2}\exp\left(\sqrt{-2\omega }L\right),$$

with const>0, where δ is the phase shift between the solitons [103]. Thus, for the fundamental solitons with S=0, Equation (39) predicts the attractive interaction between the in-phase solitons (δ=0) and repulsion between out-of-phase ones (δ=π). Accordingly, the interplay of the repulsive interaction with the effective Peierls–Nabarro potential, which is pinning the soliton to the underlying lattice [25], produces *stable* bound states of two or several mutually out-of-phase solitons, while the in-phase bound states are unstable. These predictions were confirmed by numerical results [78]. As an example, Figure 4 displays a numerically found stable bound state in the form of a string of three fundamental solitons with alternating phases.

**Figure 5:**
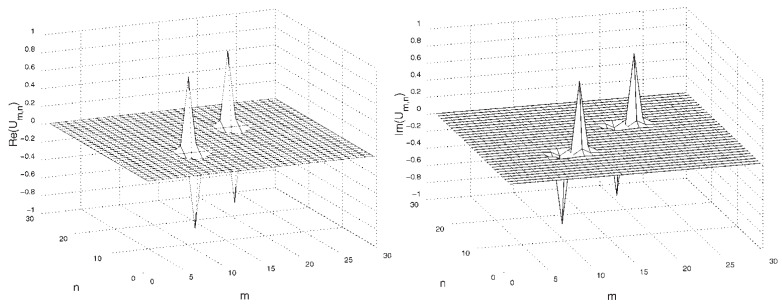
An example of a stable bound state of two in-phase discrete vortex solitons, with topological charge S=1. The result is produced by the numerical solution of Equation (38) with ω=−1. The figure is borrowed from Ref. [78].

For the pair of identical vortex solitons with S=1, Equation (39) predicts the opposite result, viz. the repulsive interaction and stability of the ensuing bound states for in-phase vortices (δ=0), and the attraction leading to the instability of the bound state in the case of δ=π. These predictions were also corroborated by numerical findings [78]; see an example of a stable bound state of two identical vortex solitons in Figure 5.

### 2.3. Two-Dimensional Discrete Solitons in Mini-Gaps of a Spatially Modulated Lattice

A specific class of self-trapped modes is gap solitons, which may populate finite bandgaps in the linear spectra of various nonlinear systems originating in optics and BECs [4,104,105]. While, in most cases, gap solitons are predicted theoretically and created experimentally in the context of matter waves [106] and optical pulses [107] in the continuum, they may naturally appear as discrete modes in *mini-gaps*, which are induced in the linear spectrum of lattice media by superimposed periodic spatial modulations (*superlattice*).

Such a 2D lattice model was introduced in Ref. [108], based on the following DNLS equation:

(40) $$i{\dot{\psi }}_{m,n}+C_{m,n}\left(\psi_{m+1,n}+\psi_{m-1,n}\right)+K_{m,n}\left(\psi_{m,n+1}+\psi_{m,n-1}\right)+{\left|\psi_{m,n}\right|}^{2}\psi_{m,n}=0,$$

where the horizontal and vertical coupling constants are modulated as follows:

(41) $$C_{m,n}=1+\Delta \cos\left(Qm\right),\;K_{m,n}=1+\Delta \cos\left(Qn\right);$$

cf. Equation (3). The superlattice represented by Equations (40) and (41) can be created by means of the technique used for making OLs in experiments with BECs [108].

**Figure 6:**
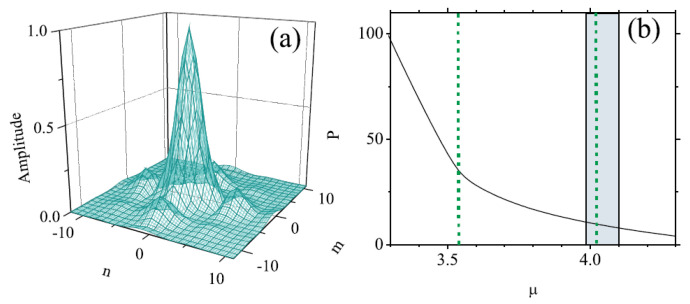
(**a**) An example of a stable 2D discrete soliton with chemical potential μ=4.05 (in this figure, the notation for the chemical potential is μ, instead of ω, adopted in the text), which corresponds to the right vertical line in (**b**), found in the mini-gap of the system based on Equations (40) and (41) with Δ=0.5 and Q=π/3. (**b**) The dependence P(μ) of the norm of the solitons populating the mini-gap, which is identical to the interval of values of μ represented in the panel. The solitons are stable in the narrow shaded interval (note that the well-known necessary (but not sufficient) Vakhitov–Kolokolov stability criterion for solitons is dP/dμ<0 [109]). The figure is borrowed from Ref. [108].

Looking for solutions in the usual form, $\psi_{m,n}\left(t\right)=\exp\left(-i\omega t\right)u_{m,n}$, with real frequency (chemical potential) ω, the numerical analysis produces the linear spectrum of this system, including the usual semi-infinite bandgap and a pair of additional narrow mini-gaps. Further, a family of fundamental 2D discrete solitons populating the mini-gaps was furnished by the numerical solution of the full nonlinear system, being stable in a small section of the mini-gap, as shown in Figure 6b. The stable 2D soliton, displayed in Figure 6a, features a typical shape of gap solitons, with a number of small satellite peaks surrounding the tall central one [105]. Bound states of two and four fundamental solitons were found as well, featuring weak instability [108].

### 2.4. Two-Dimensional Discrete Solitons in a Rotating Lattice

The dynamics of BECs loaded in OLs rotating at angular velocity Ω, as well as the propagation of light in a twisted nonlinear photonic crystal with pitch Ω, is modeled by the 2D version of Equation (1) including the lattice potential, with depth ε and period 2π/k, written in the rotating reference frame:

(42) $$i\psi_{t}=-\left[\left(1/2\right)\nabla^{2}+\Omega {\hat{L}}_{z}\right]\psi -\varepsilon \left[\cos\left(kx\right)+\cos\left(ky\right)\right]\psi +\sigma {\left|\psi \right|}^{2}\psi ,$$

where ${\hat{L}}_{z}=i\left(x\partial_{y}-y\partial_{x}\right)\equiv i\partial_{\theta }$ is the operator of the *z*-component of the orbital momentum, θ being the angular coordinate in the $\left(x,y\right)$ plane. In the tight-binding approximation, Equation (42) is replaced by the following variant of the DNLS equation [110]:

(43) $$\begin{array}{c} i{\dot{\psi }}_{m,n}=-\left(C/2\right)\left\{\left(\psi_{m+1,n}+\psi_{m-1,n}+\psi_{m,n+1}+\psi_{m,n-1}-4\psi_{m,n}\right)\right.\\ \left.-i\Omega \left[m\left(\psi_{m,n+1}-\psi_{m,n-1}\right)-n\left(\psi_{m+1,n}-\psi_{m-1,n}\right)\right]\right\}+\sigma {\left|\psi_{m,n}\right|}^{2}\psi_{m,n}\;, \end{array}$$

where *C* is the intersite coupling constant. In Ref. [110], stationary solutions to Equation (43) were looked for in the usual form (29), fixing ω≡−1 and varying *C* in (43) as a control parameter. Two species of localized states were thus constructed: off-axis fundamental discrete solitons, placed at distance *R* from the origin, and on-axis (R=0) vortex solitons, with topological numbers S=1 and 2. At a fixed value of rotation frequency Ω, a stability interval for the fundamental soliton, $0<C<C_{\max}^{\left(\mathrm{fund}\right)}\left(R\right)$, monotonously shrinks with the increase of *R*, i.e., most stable are the discrete fundamental solitons with the center placed at the rotation pivot. Vortices with S=1 are gradually destabilized with the increase of Ω (i.e., their stability interval, $0<C<C_{\max}^{\left(\mathrm{vort}\right)}\left(\Omega \right)$, shrinks). On the contrary, a remarkable finding is that vortex solitons with S=2, which, as said above, are completely unstable in the usual DNLS equation with Ω=0, are *stabilized* by the rotation, in an interval $0<C<C_{\mathrm{cr}}^{\left(S=2\right)}\left(\Omega \right)$, with $C_{\mathrm{cr}}^{\left(S=2\right)}\left(\Omega \right)$ growing as a function of Ω. In particular, $C_{\mathrm{cr}}^{\left(S=2\right)}\left(\Omega \right)\approx 2.5\Omega$ at small Ω [110].

### 2.5. Spontaneous Symmetry Breaking of the 2D Discrete Solitons in Linearly Coupled Lattices

A characteristic feature of many nonlinear *dual-core* systems, built of two identical linearly coupled waveguides with intrinsic self-attractive nonlinearity, is a *spontaneous-symmetry-breaking* (SSB) bifurcation, which destabilizes the symmetric ground state, with equal components of the wave function in the coupled cores, and creates stable asymmetric states. The SSB bifurcation takes place at a critical value of the nonlinearity strength, the asymmetric state existing above this value [111]. A system of linearly coupled DNLS equations is a basic model for SSB in discrete settings. Its 2D form is [20]

(44) $$\begin{array}{ccc} i{\dot{\phi }}_{m,n} & = & -\left(1/4\right)\left(\phi_{m+1,n}+\phi_{m-1,n}+\phi_{m,n+1}+\phi_{m,n-1}-4\phi_{m,n}\right)\\ & & -{\left|\phi_{m,n}\right|}^{2}\phi_{m,n}-K\psi_{m,n}\;,\\ i{\dot{\psi }}_{m,n} & = & -\left(1/4\right)\left(\psi_{m+1,n}+\psi_{m-1,n}+\psi_{m,n+1}+\psi_{m,n-1}-4\psi_{m,n}\right)\\ & & -{\left|\psi_{m,n}\right|}^{2}\psi_{m,n}-K\phi_{m,n}\; \end{array}$$

where $\phi_{m,n}$ and $\psi_{m,n}$ are the wave functions of the discrete coordinates *m* and *n* and K>0 represents the linear coupling between the cores. Stationary states with frequency ω are looked for as $\left(\phi_{m,n},\psi_{m,n}\right)=\exp\left(-i\omega t\right)\left(u_{m,n},v_{m,n}\right)$. Real stationary fields in the two components are characterized by their norms:

(45) $$E_{u,v}=\sum_{m,n=-\infty }^{+\infty }\left(u_{m,n}^{2},v_{m,n}^{2}\right),$$

and the asymmetry of the symmetry-broken state is determined by the parameter

(46) $$r=\left(E_{u}-E_{v}\right)/\left(E_{u}+E_{v}\right).$$

**Figure 7:**
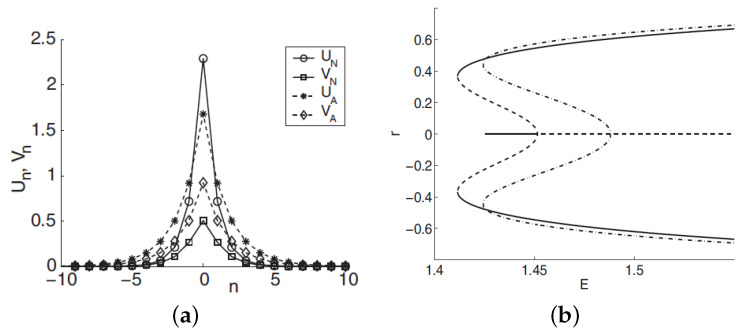
(**a**) A stable 2D two-component discrete soliton with spontaneously broken symmetry between the components, generated by the system (44). The 2D soliton, with total norm $E\equiv E_{u}+E_{v}=1.435$, is displayed by means of its cross-section. Symbols labeled $\left(U_{N},V_{N}\right)$ and $\left(U_{A},V_{A}\right)$ stand, respectively, for the components of the numerically constructed soliton and its analytical counterpart predicted by the VA based on the ansatz (47). (**b**) Families of 2D on-site-centered discrete solitons, generated by the system (44), are shown by means of curves r(E), where *r* is the asymmetry parameter (46). The dashed–dotted curve shows the VA prediction, while the solid and dashed ones represent stable and unstable solitons produced by the numerical solution. The figure is borrowed from Ref. [20].

The system under consideration can be analyzed by means of the VA based on the 2D ansatz:

(47) $$\left(u_{m,n},v_{m,n}\right)=\left(A,B\right)\exp\left[-a\left(|m|+|n|\right)\right],$$

with inverse width *a* and the amplitudes, *A* and *B*, of the two components (cf. the 1D ansatz (32)). SSB is represented by solutions with A≠B. An example of a stable 2D discrete soliton is displayed in Figure 7a, which corroborates the accuracy of the VA. In Figure 7b, the families of symmetric and asymmetric 2D discrete solitons is characterized by the dependence of asymmetry parameter *r*, defined as per Equation (46), on the total norm, $E\equiv E_{u}+E_{v}$; see Equation (45). Figure 7b demonstrates the SSB bifurcation of the *subcritical* type [112], with the two branches of broken-symmetry states originally going backward in the *E* direction, as unstable ones; they become stable after passing the turning point. Accordingly, Figure 7b demonstrates a bistability area, where symmetric and asymmetric states coexist as stable ones.

## 3. Two-Dimensional Discrete Solitons in the Salerno Model (SM)

The 2D version of the SM was introduced in Ref. [113]:

(48) $$\begin{array}{ccc} i{\dot{\psi }}_{n,m} & = & -\left[\left(\psi_{n+1,m}+\psi_{n-1,m}\right)+\left(\psi_{n,m+1}+\psi_{n,m-1}\right)\right]\\ & \times & \left(1+\mu {\left|\psi_{n,m}\right|}^{2}\right)-2{\left|\psi_{n,m}\right|}^{2}\psi_{n,m}\;, \end{array}$$

Similar to its 1D version (11), Equation (48) conserves the norm and Hamiltonian (cf. Equations (12) and (14)):

(49) $${\left(N_{\mathrm{SM}}\right)}_{2D}=\left(1/\mu \right)\sum_{m,n}\ln\left|1+\mu |\psi_{n,m}{|}^{2}\right|\;,$$

(50) $$\begin{array}{c} {\left(H_{\mathrm{SM}}\right)}_{2D}=-\sum_{n,m}\left[\left(\psi_{n,m}\psi_{n+1,m}^{*}+\psi_{n+1,m}\psi_{n,m}^{*}\right)+\left(\psi_{n,m}\psi_{n,m+1}^{*}+\psi_{n,m+1}\psi_{n,m}^{*}\right)\right.\\ \left.+\left(2/\mu \right)|\psi_{n,m}{|}^{2}\right]+\left(2/\mu \right){\left(N_{\mathrm{SM}}\right)}_{2D}.\; \end{array}$$

The continuum limit of this model is the 2D equation, which is an extension of its 1D counterpart (17):

(51) $$i\Psi_{t}+\left(1+\mu {\left|\Psi \right|}^{2}\right)\left(\Psi_{xx}+\Psi_{yy}\right)+2\left(2\mu +1\right){\left|\Psi \right|}^{2}\Psi =0.$$

Note that the effective nonlinear diffraction term $\mu {\left|\Psi \right|}^{2}\left(\Psi_{xx}+\Psi_{yy}\right)$ in Equation (51) with either sign of μ prevents the onset of the collapse because, in the limit of the catastrophic self-compression, this term becomes dominant, giving a positive contribution to the energy. Thus, this term makes it possible to construct stable 2D solitons [113].

**Figure 8:**
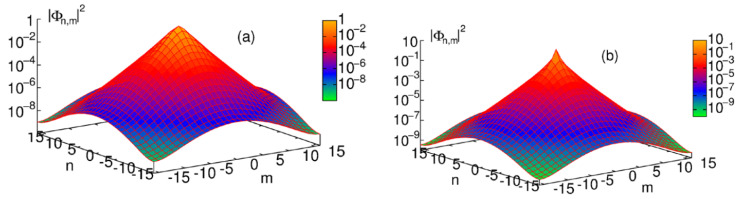
Stable discrete solitons in the 2D Salerno model with competing nonlinearities (μ<0 in Equation (48)), obtained for ω=−4.22: (**a**) a regular soliton at μ=−0.2; (**b**) a cuspon at μ=−0.88. The figure is borrowed from Ref. [113].

The 2D discrete solitons are looked for as solutions to Equation (51) in the usual form, $\psi_{m,n}\left(t\right)=e^{-i\omega t}\Phi_{m,n}$. In the most interesting case of the competing nonlinearities, μ<0, the situation is similar to that outlined above for the one-dimensional SM: there are ordinary discrete solitons, which have their stability and instability regions, and 2D cuspons, which are entirely stable in their existence region. Typical 2D solitons of both types are displayed in Figure 8. Antisymmetric bound states of ordinary 2D discrete solitons, and symmetric complexes built of 2D cuspons, are stable, while the bound states of cuspons with opposite parities are unstable, also like in the 1D model.

**Figure 9:**
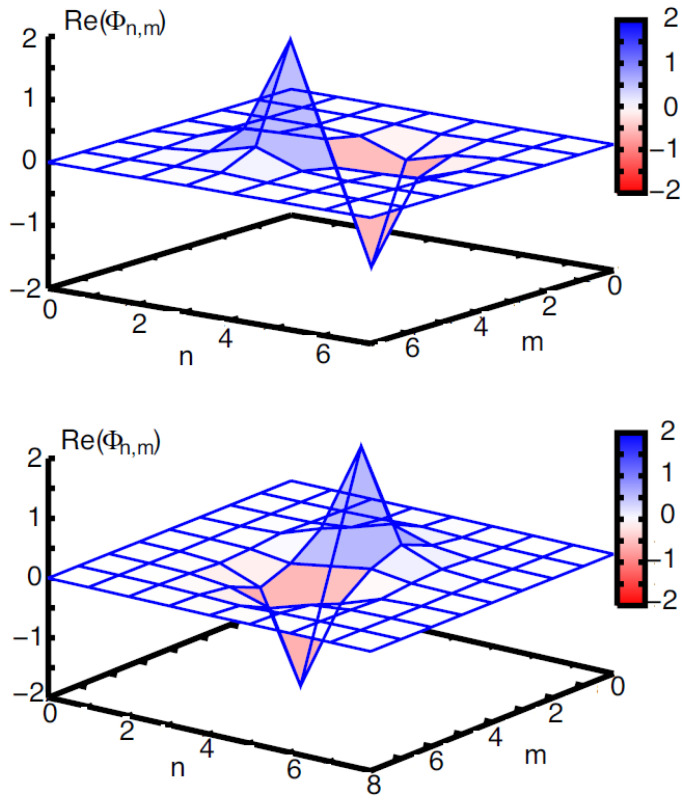
Examples of discrete vortex solitons with topological charge S=1, produced by the 2D SM, based on Equation (48). Profiles of the real part of the stationary wave function $\Phi_{m,n}$ for the vortices of the on-site-centered (stable *vortex cross*) and intersite-centered (unstable *vortex square*) types are displayed in the top and bottom panels, respectively. Both solutions are obtained for μ=−0.4 and ω=7.0. The figure is borrowed from Ref. [113].

Along with the fundamental solitons, the 2D SM with the competing nonlinearities gives rise to vortex soliton modes, which may be stable in narrow parameter regions [113]. Examples of on-site- and intersite-centered vortex solitons (alias *vortex cross* and *vortex square*, respectively) are presented in Figure 9. In the 2D SM with non-competing nonlinearities (μ>0 in Equation (51)), vortex solitons are unstable, spontaneously transforming into fundamental ones and losing their vorticity. This transition is possible because the angular momentum is not conserved in the lattice system. The situation is different in the 2D SM with competing nonlinearities (μ<0), where unstable vortex modes transform into *vortical breathers*, i.e., persistently oscillating localized modes that keep the original vorticity.

## 4. Solitons of the Semi-Vortex and Mixed-Mode Types in the Discrete 2D Spin–Orbit-Coupling System

Recently, much interest has been drawn to the emulation of the spin–orbit-coupling (SOC) effect, which is well known in the physics of semiconductors, in binary BECs [114,115,116,117]. While SOC is a linear effect, its interplay with the intrinsic mean-field nonlinearity of atomic BECs gives rise to the prediction of new species of 1D, 2D, and 3D solitons [118]. In particular, the effectively 2D binary BEC with the SOC of the *Rashba type* is modeled by the following system of coupled GP equations for two components $\phi^{\left(\pm \right)}$ of the pseudo-spinor wave function [119]:

(52) $$\begin{array}{ccc} i\frac{\partial \phi^{\left(+\right)}}{\partial t} & = & -\frac{1}{2}\nabla^{2}\phi^{\left(+\right)}-\left(\right|\phi^{\left(+\right)}{|}^{2}+\gamma |\phi^{\left(-\right)}{|}^{2})\phi^{\left(+\right)}+2\lambda \left(\frac{\partial \phi^{\left(-\right)}}{\partial x}-i\frac{\partial \phi^{\left(-\right)}}{\partial y}\right),\\ i\frac{\partial \phi^{\left(-\right)}}{\partial t} & = & -\frac{1}{2}\nabla^{2}\phi^{\left(-\right)}-\left(\right|\phi^{\left(-\right)}{|}^{2}+\gamma |\phi^{\left(+\right)}{|}^{2})\phi^{\left(-\right)}+2\lambda \left(-\frac{\partial \phi^{\left(+\right)}}{\partial x}-i\frac{\partial \phi^{\left(+\right)}}{\partial y}\right). \end{array}$$

In this system, the SOC is represented by coefficient λ, which couples the two equations through the first-order spatial derivatives. The system also includes the self- and cross-attractive nonlinearities, with scaled coefficients 1 and γ, respectively.

The system of coupled GP equations (52) maintains 2D solitons of two different types, namely semi-vortices (SVs) and mixed modes (MMs) [119]. The SV solitons, written in polar coordinates $\left(r,\theta \right)$, have zero vorticity in one component and vorticity +1 or −1 in the other:

(53) $$\begin{array}{ccc} \phi_{1}^{\left(+\right)} & = & e^{-i\omega t}f_{1}\left(r^{2}\right),\;\phi_{1}^{\left(-\right)}=e^{-i\omega t+i\theta }rf_{2}\left(r^{2}\right), \end{array}$$

(54) $$\begin{array}{ccc} \phi_{2}^{\left(+\right)} & = & e^{-i\omega t-i\theta }rf_{2}\left(r^{2}\right),\;\phi_{2}^{\left(-\right)}=e^{-i\omega t}f_{1}\left(r^{2}\right), \end{array}$$

where ω is the chemical potential and $f_{1,2}\left(r^{2}\right)$ are real functions, which take finite values at r=0 and exponentially decay $∼\left(\sin\left(2\lambda r\right),\cos\left(2\lambda r\right)\right)\exp\left(-\sqrt{2\left(-\omega -2\lambda^{2}\right)}r\right)$ at r→∞. The two SV solutions (53) and (54), which are mirror images of each other, exist in the semi-infinite bandgap, $\omega <-2\lambda^{2}$.

The combination of zero and nonzero vorticities in the SV solutions (53) and (54) is exactly compatible with the structure of the coupled GP equations (52). Contrary to this, MM solitons cannot be represented by an exact ansatz similar to Equations (53) and (54), but they may be approximated by a linear combination of both types of the SVs, $\left\{\phi_{1}^{\left(+\right)}+\phi_{2}^{\left(+\right)},\phi_{1}^{\left(-)(1\right)}+\phi_{2}^{\left(+\right)}\right\}$. An essential result is that the SVs and MMs are stable and represent the system’s ground state in the cases of γ<1 and γ>1, respectively, i.e., when the self-attraction is stronger or weaker than the cross-attraction in Equations (52) [119]. On the other hand, the SVs and MMs are unstable, as excited states, in the opposite cases, i.e., γ>1 and γ<1, respectively.

The discretized version of the SOC GP system (52), which corresponds to the spin–orbit-coupled binary BEC trapped in a deep OL potential, with discrete coordinates (m,n), was introduced in Ref. [120]:

(55) $$\begin{array}{c} i\frac{\partial \phi_{m,n}^{\left(+\right)}}{\partial t}=-\frac{1}{2}\left(\phi_{m+1,n}^{\left(+\right)}+\phi_{m-1,n}^{\left(+\right)}+\phi_{m,n+1}^{\left(+\right)}+\phi_{m,n-1}^{\left(+\right)}-4\phi_{m,n}^{\left(+\right)}\right)\\ -\left(\right|\phi_{m,n}^{\left(+\right)}{|}^{2}+\gamma |\phi_{-}{|}^{2})\phi_{m,n}^{\left(+\right)}+\lambda \left[\phi_{m+1,n}^{\left(-\right)}-\phi_{m-1,n}^{\left(-\right)}-i\left(\phi_{m,n+1}^{\left(-\right)}-\phi_{m,n-1}^{\left(-\right)}\right)\right],\\ i\frac{\partial \phi_{m,n}^{\left(-\right)}}{\partial t}=-\frac{1}{2}\left(\phi_{m+1,n}^{\left(-\right)}+\phi_{m-1,n}^{\left(-\right)}+\phi_{m,n+1}^{\left(-\right)}+\phi_{m,n-1}^{\left(-\right)}-4\phi_{m,n}^{\left(-\right)}\right)\\ -\left(\right|\phi_{m,n}^{\left(-\right)}{|}^{2}+\gamma |\phi_{m,n}^{\left(+\right)}{|}^{2})\phi_{-}+\lambda \left[-\left(\phi_{m+1,n}^{\left(+\right)}-\phi_{m-1,n}^{\left(+\right)}\right)-i\left(\phi_{m,n+1}^{\left(+\right)}-\phi_{m,n-1}^{\left(+\right)}\right)\right]. \end{array}$$

The linearized version of this system gives rise to the following dispersion relation for the discrete plane waves, $\phi_{m,n}^{\left(\pm \right)}∼\exp\left(-i\omega t+ipx+iqy\right)$, with the wavenumbers taking values in the first Brillouin zone, 0<p,q<2π:

(56) $$\omega =2\left(\sin^{2}\frac{p}{2}+\sin^{2}\frac{q}{2}\right)\pm 2\lambda \sqrt{\sin^{2}p+\sin^{2}q}.$$

**Figure 10:**
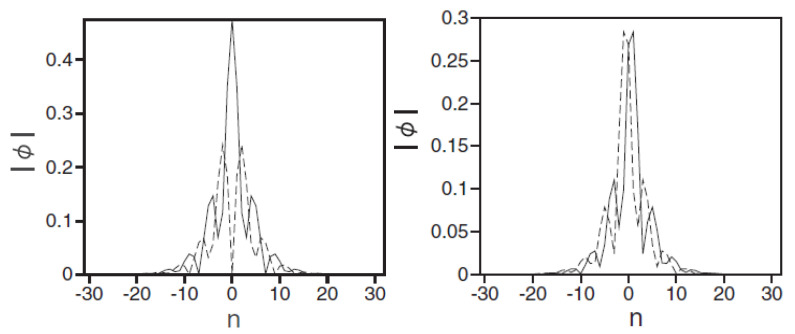
(**Left**) Juxtaposed profiles of $\left|\phi_{m,n}^{\left(+\right)}\right|$ and $\left|\phi_{m,n}^{\left(-\right)}\right|$ (solid and dashed lines, respectively) of a stable 2D discrete soliton of the semi-vortex type, in the central cross-section, produced by a numerical solution of Equation (55) with λ=0.53 and γ=0. The soliton’s norm (see Equation (57)) is N=3.5. (**Right**) The same for a stable discrete soliton of the mixed-mode type, with λ=0.58, γ=2, and N=2. Values of the discrete fields at lattice sites are connected by lines, for better visualization. The figure is borrowed from Ref. [120].

The numerical solution of Equation (55) has produced 2D modes, which are discrete counterparts of the SV and MM solitons of the continuum system (52); see the examples in Figure 10. As concerns the stability, the discreteness extends the stability of the SV and MM solitons towards γ>1 and γ<1, respectively.

**Figure 11:**
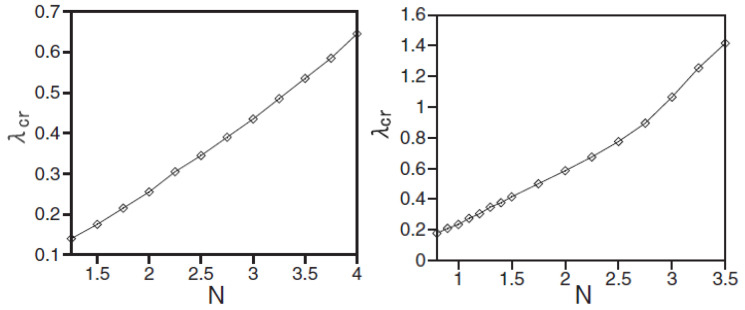
(**Left** and **right**) The dependence of the critical value of the SOC strength, $\lambda_{\mathrm{cr}}$, above which the 2D discrete solitons of the SV and MM types, produced by the numerical solution of Equation (55) with γ=0 and 2, respectively, suffer the delocalization, on the total soliton’s norm (57). The figure is borrowed from Ref. [120].

A drastic difference of the discrete solitons of both the SV and MM types from their counterparts in the continuum is that they suddenly suffer delocalization (decay) when the SOC strength λ in Equation (55) exceeds a certain critical value, $\lambda_{\mathrm{cr}}$. The dependence of $\lambda_{\mathrm{cr}}$ on the soliton’s norm:

(57) $$N=\sum_{m,n}\left({\left|\phi_{m,n}^{\left(+\right)}\right|}^{2}+{\left|\phi_{m,n}^{\left(-\right)}\right|}^{2}\right),$$

for the SV and MM solitons is displayed in Figure 11. The onset of the delocalization may be explained as a transition of the solution from the spectral bandgap to the band populated by the small-amplitude plane-wave states in the system’s linear spectrum, which is produced by Equation (56).

## 5. Stable Soliton Species in the 3D Discrete Nonlinear Schrödinger Equation

### 5.1. The 3D Setting

A natural development of the analysis of the solitons and solitary vortices, and their bound states, produced by the 2D discrete DNLS equation and its extensions, which is outlined above in Section 2, Section 3 and Section 4, is to construct self-trapped states (solitons) in the framework of the 3D equation:

(58) $$\begin{array}{c} i{\dot{\phi }}_{l,m,n}+C\left(\phi_{l+1,m,n}+\phi_{l,m+1,n}+\phi_{l,m,n+1}+\phi_{l-1,m,n}+\phi_{l,m-1,n-1}+\phi_{l,m,n-1}-6\phi_{l,m,n}\right)\\ +{\left|\phi_{l,m,n}\right|}^{2}\phi_{l,m,n}=0, \end{array}$$

where, as above, the overdot stands for the time derivative, $\left(l,m,n\right)$ is the set of the 3D discrete coordinates, and C>0 is the coefficient of the intersite coupling. The 3D DNLS equation cannot be realized in optics, but it admits a natural implementation for BECs loaded in a deep 3D OL potential [4,105,121]. In that case, $\phi_{l,m,n}\left(t\right)$ is the the respective effectively discretized BEC wave function.

As above, stationary soliton solutions to Equation (58) with chemical potential ω are looked for as

(59) $$\phi_{l,m,n}=\exp\left(-i\omega t\right)u_{l,m,n},$$

where the stationary discrete wave function $u_{l,m,n}$ obeys the corresponding equation:

(60) $$\begin{array}{c} \omega u_{l,m,n}+C\left(u_{l+1,m,n}+u_{l,m+1,n}+u_{l,m,n+1}+u_{l-1,m,n}+u_{l,m-1,n-1}+u_{l,m,n-1}-6u_{l,m,n}\right)\\ +{\left|u_{l,m,n}\right|}^{2}u_{l,m,n}=0. \end{array}$$

In particular, numerical solutions of Equation (60) for 3D discrete solitons with embedded vorticity S=0,1,2,... (S=0 corresponds to the fundamental solitons, for which the wave function $u_{l,m,n}$ is real) can be obtained, starting from the natural input:

(61) $$u_{l,m,n}^{\left(\mathrm{init}\right)}=A{\left(l+im\right)}^{S}\mathrm{sech}\left(\eta \sqrt{l^{2}+m^{2}}\right)\exp\left(-|n|\right),$$

where η is a real scale parameter, and it is implied that the vorticity axis is directed along the coordinate *n* [122].

It is also relevant to consider a two-component system of nonlinearly coupled 3D DNLS equations, for wave functions $\phi_{l,m,n}\left(t\right)$ and $\psi_{l,m,n}\left(t\right)$ of two interacting BEC species (most typically, these are different hyperfine states of the same atom) [122]:

(62) $$\begin{array}{c} i{\dot{\phi }}_{l,m,n}+C\left(\phi_{l+1,m,n}+\phi_{l,m+1,n}+\phi_{l,m,n+1}+\phi_{l-1,m,n}+\phi_{l,m-1,n-1}+\phi_{l,m,n-1}-6\phi_{l,m,n}\right)\\ +\left({\left|\phi_{l,m,n}\right|}^{2}+\beta {\left|\psi_{l,m,n}\right|}^{2}\right)\phi_{l,m,n}=0,\\ i{\dot{\psi }}_{l,m,n}+C\left(\psi_{l+1,m,n}+\psi_{l,m+1,n}+\psi_{l,m,n+1}+\psi_{l-1,m,n}+\psi_{l,m-1,n-1}+\psi_{l,m,n-1}-6\psi_{l,m,n}\right)\\ +\left({\left|\psi_{l,m,n}\right|}^{2}+\beta {\left|\phi_{l,m,n}\right|}^{2}\right)\psi_{l,m,n}=0, \end{array}$$

Here, β>0 is the relative strength of the inter-component attractive interaction with respect to the intra-component self-attraction.

### 5.2. Results

#### 5.2.1. Single-Component 3D Solitons

The numerical analysis, starting from input (61), has provided families of fundamental and vortex solitons. Here, following Ref. [122], the results are displayed for a fixed value of the chemical potential, ω=−2 in Equation (59), while varying the coupling constant *C* in Equations (58) and (60). In particular, the discrete fundamental solitons with S=0 are stable at C<$C_{\mathrm{cr}}^{\left(0\right)}\approx 2$, and the vortex modes with S=1 are stable at C<$C_{\mathrm{cr}}^{\left(1\right)}\approx 0.65$. Note that the limit of C→∞ corresponds to the 3D NLS equation in the continuum, in which all solitons are definitely unstable; therefore, all discrete solitons become unstable at sufficiently large values of *C*. At $C>C_{\mathrm{cr}}^{\left(1\right)}$, the simulations demonstrate that the development of the instability destroys the vortical structure and, eventually, transforms the soliton into a fundamental one, with S=0 (not shown here in detail). The change of the topological charge is possible, as the angular momentum is not a dynamical invariant of the lattice dynamics.

**Figure 12:**
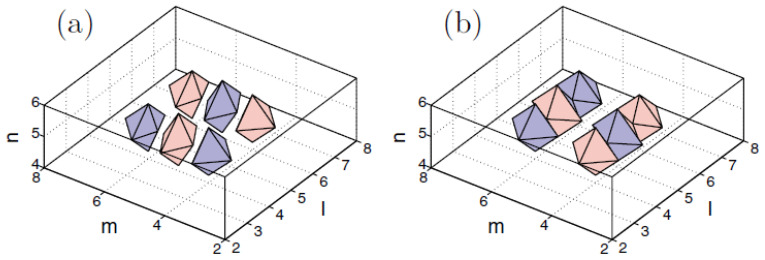
A stable discrete vortex soliton with topological charge S=3, produced by the numerical solution of Equation (60) with C=0.01 and ω=−2. Panels (**a**) and (**b**) display level contours corresponding to Re$\left(u_{l,m,n}\right)=\pm 0.25$ and Im$\left(u_{l,m,n}\right)=\pm 0.25$, respectively. The figure is borrowed from Ref. [122].

The vortex solitons with S=2 are completely unstable, but an unusual feature of these states is that, at sufficiently small values of *C* (in particular, at C=0.01), the instability spontaneously rebuilds them into stable discrete solitons with a *larger vorticity*, S=3 [122]. An example of a stable soliton with S=3 is displayed in Figure 12.

**Figure 13:**
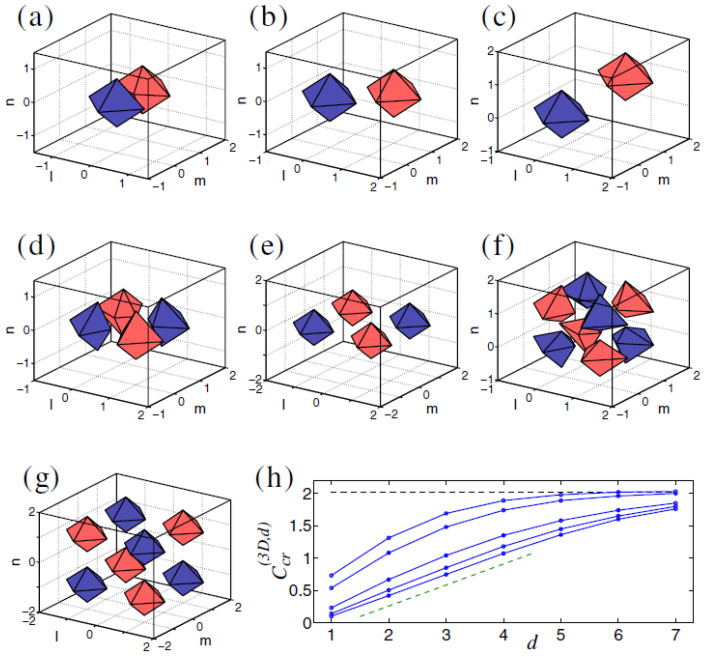
Stable 3D multipole solutions of Equation (60) with C=0.1 and ω=−2. The top row depicts stable tightly bound dipoles (with intersite separation d=1): (**a**) straight, (**b**) 2D-diagonal, and (**c**) 3D-diagonal ones. (**d**,**e**) Quadrupoles set in the n=0 plane, with intrinsic separation d=1 and d=2, respectively. (**f**,**g**) Octupoles with d=1 and d=2. Panel (**h**) displays the stability boundary $C_{cr}^{\left(3D,d\right)}$ as a function of the intrinsic separation *d* for diagonal, oblique, and straight dipoles, octupoles, and quadrupoles, from top to bottom. The horizontal dashed line designates the stability threshold for the fundamental discrete soliton. Note that, for the quadrupoles (the bottom boundary), $C_{\mathrm{quad}}^{\left(3D,d\right)}$ is a linear function of *d* at d≤4 (see the dashed straight line with slope 0.325, included for guidance). In panels (**a**–**g**), level contours corresponding to Re(ul,m,n)=±0.5 are shown by blue and red (colors, respectively). The figure is borrowed from Ref. [123].

In addition to the fundamental and vortex solitons, Equations (58) and (60) produce diverse multimode species of stable discrete 3D solitons in the form of dipoles, quadrupoles, and octupoles [123]. Examples of such states are presented in Figure 13 for C=0.1. This figure displays tightly bound dipoles with different orientations with respect to the lattice, viz. straight, 2D-diagonal, and 3D-diagonal ones (panels (a,b,c)), quadrupole (panel (d)), and octupole (panel (f)), in which the field fills adjacent sites of the lattice (with the lattice distance between them d=1). Also displayed are loosely bound quadrupole and octupole (in panels (e) and (g), respectively), with distance d=2 between the filled (excited) sites. Similar multimode states with still larger separations *d* between the filled sites were found as well. The results are summarized in Figure 13h, which shows the stability boundaries $C_{cr}^{\left(3D,d\right)}$ for dipoles, quadrupoles and octupoles vs. *d*. Naturally, the stability region, $C<C_{cr}^{\left(3D,d\right)}$, increases with the increase of *d*, as the interaction between the filled sites, which leads to the possible dynamical instability of the multipole states, is weaker for larger *d*.

**Figure 14:**
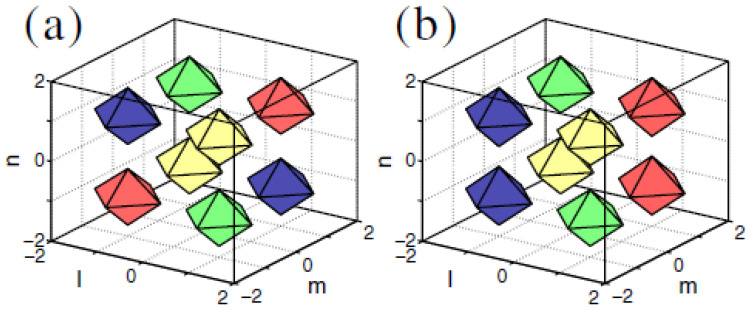
Vortex cubes produced by the numerical solution of Equation (60) with Λ=2 and C=0.1. Panel (**a**) shows a stable composite mode, built of two parallel identical quasi-planar vortices with topological numbers $S_{1}=S_{2}=1$ and a phase shift of π. Panel (**b**) shows an unstable vortex–antivortex cube, formed by vortices with opposite topological charges, $S_{1}=-S_{2}$. Level contours corresponding to Re(ul,m,n)=±0.5 are shown by blue and red colors, and the contours corresponding to Im(ul,m,n)=±0.5 are shown by green and yellow colors, respectively. The figure is borrowed from Ref. [123].

In addition to the above-mentioned states, Equation (60) admits more-sophisticated stable composite states, such as “vortex cubes”, built as a pair of identical parallel quasi-planar vortices with topological numbers $S_{1}=S_{2}=1$ and opposite signs (the phase shift π), set in parallel planes, as shown in Figure 14a. Stationary solutions representing vortex–antivortex cubes, in the form of parallel quasi-planar vortices with opposite topological charges, $S_{1}=-S_{2}=1$, can be found as well, as shown in Figure 14b, but they are completely unstable.

The same Equation (58) gives rise to other stable self-trapped modes, such as vortex solitons with the axis directed along the 2D diagonal, cf. Figure 13b. Vortex modes with the axis parallel to the 3D diagonal exist as well, but they are unstable; see further details in Ref. [123].

#### 5.2.2. Two-Component 3D Solitons (Including Skyrmions)

**Figure 15:**
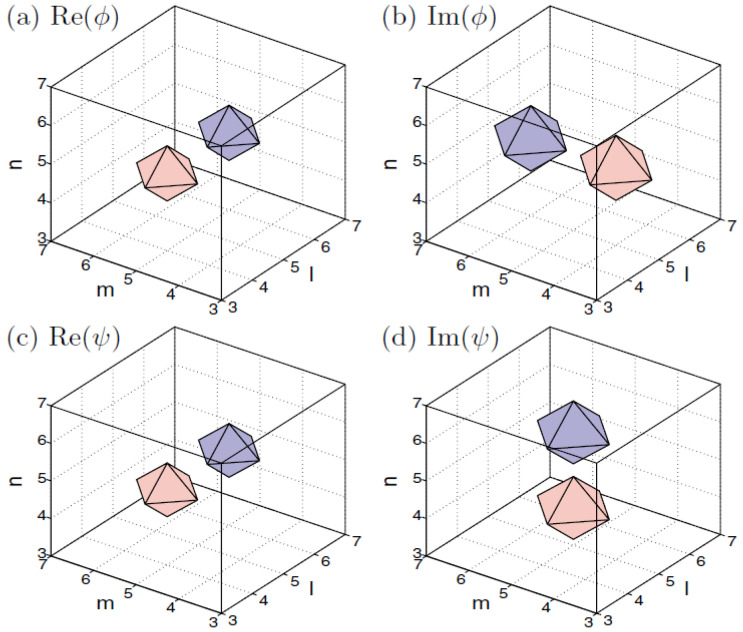
A stable bound state of two mutually orthogonal vortex solitons with topological charges S=−1. The bound state is obtained as a numerical solution of Equations (62) with β=0.5, C=0.01, and the chemical potentials of both components ω=−2. Top and bottom panels represent, respectively, the first and second components of the system. The panels show the contours of Re(ul,m,n)=±0.5 and Im(ul,m,n)=±0.5. The bluish and reddish colors designate the positive and negative values, respectively. The figure is borrowed from Ref. [122].

The system of coupled 3D DNLS equations (62) produces stable soliton states, which are specific to the two-component nonlinear lattice medium. A noteworthy example is a composite mode built as a bound state of vortex solitons in the two components with *mutually orthogonal* orientations; see an example in Figure 15. These bound states are stable for sufficiently small values of the coupling constant, such as C=0.01 in Figure 15, and for β<1 in Equation (62), i.e., under the condition that the self-attraction in each component is stronger than the inter-component attraction.

**Figure 16:**
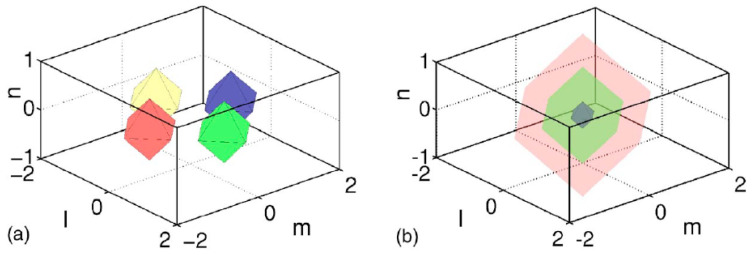
An example of a 3D discrete skyrmion, produced by the numerical solution of Equations (63) with C=0.05, β=0.25, and ω=2. Panels (**a**) and (**b**) show, respectively, the contours of the complex field, corresponding to Re(u)=+1/−1 (blue/red colors) and Im(u)=+1/−1 (green/yellow colors), and of the real field, corresponding to v=(1,0,−1) (blue, green, and red colors, designating the contours from the inside out). The figure is borrowed from Ref. [127].

The system of coupled GP equations with the repulsive sign of the nonlinearity may be used as the simplest model producing skyrmions in the binary BEC [124,125,126]. The discretization of the GP system leads to Equations (62) with the opposite sign in front of the nonlinear terms [127]. Then, these equations are reduced to stationary ones by the usual substitution with the chemical potential ω, $\left\{\phi ,\psi \right\}=\exp\left(-i\omega t\right)\left\{u_{l,m,n},v_{l,m,n}\right\}$:

(63) $$\begin{array}{c} C\left(u_{l+1,m,n}+u_{l,m+1,n}+u_{l,m,n+1}+u_{l-1,m,n}+u_{l,m-1,n-1}+u_{l,m,n-1}-6u_{l,m,n}\right)\\ -\left({\left|u_{l,m,n}\right|}^{2}+\beta {\left|v_{l,m,n}\right|}^{2}\right)u_{l,m,n}=-\omega u_{l,m,n}\;,\\ C\left(v_{l+1,m,n}+v_{l,m+1,n}+v_{l,m,n+1}+v_{l-1,m,n}+v_{l,m-1,n-1}+v_{l,m,n-1}-6v_{l,m,n}\right)\\ -\left({\left|v_{l,m,n}\right|}^{2}+\beta {\left|u_{l,m,n}\right|}^{2}\right)v_{l,m,n}=-\omega v_{l,m,n}\;, \end{array}$$

where the relative strength β of the inter-component repulsion with respect to the self-repulsion remains a positive coefficient. For ω>0, skyrmions can be constructed by choosing the field $u_{l,m,n}$ as a complex one, representing a quasi-flat vortex soliton with topological charge S=1, and the *real field* $v_{l,m,n}$ as a *bubble* into which the vortex soliton is embedded, with a nonzero background value at $\left(|l|,|m|,|n|\right)\to \infty$, viz. $v_{\mathrm{background}}^{2}=\omega$ [127]. An example of a numerically found skyrmion solution of this type is displayed in Figure 16.

The same work [127] reported solutions for 2D discrete “baby skyrmions”, which are the lattice counterparts of the modes produced by the 2D reduction of the Skyrme model [128,129]. They have a simple structure similar to their 3D counterpart displayed in Figure 16, i.e., a complex 2D vortex soliton in one component, embedded into a bubble of the delocalized field in the other real component.

## 6. Two-Dimensional Solitons and Solitary Vortices in Semi-Discrete Systems

### 6.1. Spatiotemporal Optical Solitons in Arrayed Waveguides

The consideration of 2D and 3D settings suggests a natural option to introduce 2D semi-discrete systems, with a continuous coordinate in one direction and a discrete coordinate in another, as well as 3D systems, where one or two coordinates are continuous, while the remaining one or two coordinates are discrete. In optics, a well-known 2D setting belonging to this class represents the spatiotemporal propagation of light in an array of optical fibers [130]. It is modeled by the system of linearly coupled NLS equations for complex amplitudes $u_{n}\left(z,\tau \right)$ of optical fields in individual fibers:

(64) $$i\partial_{z}u_{n}+\left(1/2\right)D\partial_{\tau }^{2}u_{n}+\left(\kappa /2\right)\left(u_{n+1}+u_{n-1}-2u_{n}\right)+{\left|u_{n}\right|}^{2}u_{n}=0,$$

where *z* is the propagation distance, $\tau \equiv t-x/V_{\mathrm{gr}}$ (with time *t* and carrier group velocity $V_{\mathrm{gr}}$) is the usual temporal variable, real *D* is the group-velocity-dispersion coefficient in each fiber, κ>0 is the coefficient of coupling between adjacent fibers in the array, and the nonlinearity coefficient is normalized to be 1. It is commonly known that optical solitons (semi-discrete ones, in the present case) can be supported in the case of anomalous dispersion, i.e., D>0.

**Figure 17:**
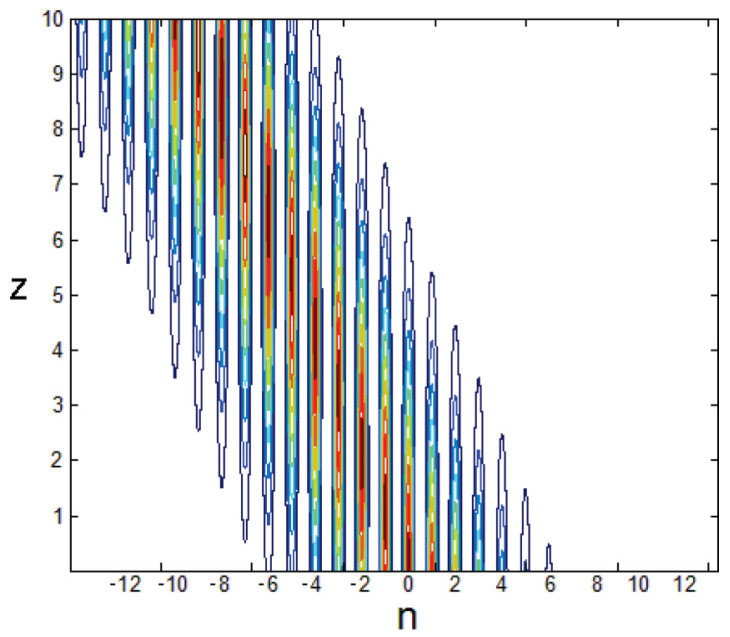
An example of a semi-discrete localized spatiotemporal mode, generated by Equation (64), which performs stable transverse motion under the action of the kick, defined according to (65), with a=1.5. The cross-section of the plot at any fixed *z* shows the distribution of power ${\left|u_{n}\left(\tau \right)\right|}^{2}$ for each *n*. The figure is borrowed from Ref. [81].

A remarkable counter-intuitive property of semi-discrete localized modes generated by Equation (64) is their ability to stably move *across the array*, under the action of a kick applied at z=0 [81]:

(65) $$u_{n}\left(\tau \right)\to \exp\left(ian\right)u_{n}\left(\tau \right),$$

with real *a*, in spite of the presence of the respective quasi-1D Peierls–Nabarro potential. An example of such a moving mode is displayed in Figure 17. This property may be compared to the above-mentioned mobility of 1D discrete solitons in the DNLS equation [45] and of 2D discrete solitons in the framework of the $\chi^{\left(2\right)}$ system (25).

**Figure 18:**
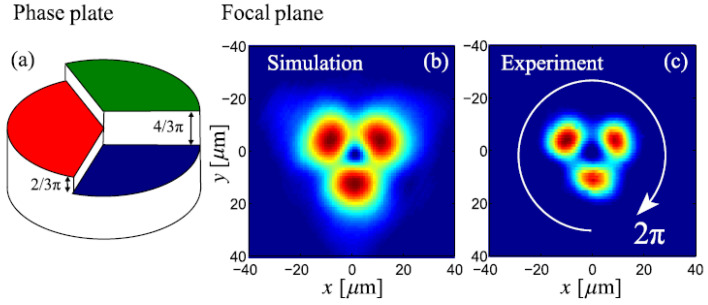
A semi-discrete vortex soliton in a hexagonal quasi-discrete array of waveguides made in bulk silica. (**a**) The phase plate used for imprinting the vortex structure into the input beam. (**b**,**c**) Numerically simulated and experimentally observed (transient) intensity distributions in the transverse plane, with phase shifts 2π/3 between adjacent peaks, which represent the simplest vortical phase structure with the topological charge S=1. The figure is borrowed from Ref. [132].

Similarly, quasi-discrete settings modeled by an extension of (64) with two transverse spatial coordinates were used for the creation of spatiotemporal optical solitons (“light bullets”) [131], as well as soliton-like transient modes with embedded vorticity [132]. Waveguides employed in those experiments feature a transverse hexagonal lattice structure, written in bulk silica by means of an optical technology. A spatiotemporal vortex state observed in the bundle-like structure (in the experiment, it is actually a transient state) is represented by Figure 18, which displays both numerically predicted and experimentally observed distributions of the intensity of light in the transverse plane, together with a phase plate used in the experiment to embed the vorticity into the incident spatiotemporal pulse, which was used to create the mode.

### 6.2. Semi-Discrete Quantum and Photonic Droplets

A new type of semi-discrete solitons was recently elaborated in Ref. [83], in the framework of an array of linearly coupled 1D GP equations, including the above-mentioned Lee–Huang–Yang correction, which represents an effect of quantum fluctuations around the mean-field states of a binary BEC [31,35]. The system is

(66) $$i\partial_{t}\psi_{j}=-\left(1/2\right)\partial_{zz}\psi_{j}-\left(C/2\right)\left(\psi_{j+1}-2\psi_{j}+\psi_{j-1}\right)+g|\psi_{j}{|}^{2}\psi_{j}-\left|\psi_{j}\right|\psi_{j},$$

where $\psi_{j}\left(z\right)$ is the mean-field wave function in the *j*-th core with the coordinate *z*, *C* is the effective inter-core coupling constant, the self-attractive quadratic term represents the Lee–Huang–Yang correction in the 1D limit (cf. Equation (7)), and g>0 accounts for the mean-field self-repulsion.

A semi-discrete system similar to the one modeled by Equation (66), but with the cubic–quintic nonlinearity instead of the combination of the quadratic and cubic terms in Equation (66), was derived in the context of nonlinear optics [83]:

(67) $$i\partial_{z}u_{n}=-\left(1/2\right)\partial_{xx}u_{n}-\left(C/2\right)\left(u_{n+1}-2u_{n}+u_{n-1}\right)-|u_{n}{|}^{2}u_{n}+{\left|u_{n}\right|}^{4}u_{n}.$$

**Figure 19:**
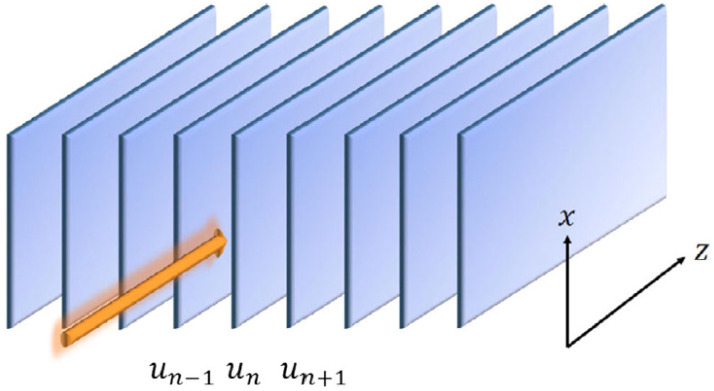
The realization of the semi-discrete system (67): the array of planar optical waveguides (blue slabs), separated by isolating layers, with the continuous transverse coordinate, *x*, and the discrete one, *n*. As shown by the arrow, light is coupled into the array along the *z* direction. The figure is borrowed from Ref. [83].

It corresponds to the array of parallel-coupled planar waveguides, as shown in Figure 19. In this case, $u_{n}\left(x,z\right)$ is the complex local amplitude of the optical wave in the *n*-th waveguide, *z* is the propagation distance, and *x* is the transverse coordinate in each waveguide, while *C* is the effective coupling constant, similar to Equation (66). By analogy with the quantum droplets, semi-discrete solitons produced by Equation (67) may be called “photonic droplets”.

The droplets produced by Equations (66) and (67) are characterized by the total norm, which is proportional to the number of atoms in the BEC:

(68) $$N=\sum_{j}\int_{-\infty }^{+\infty }{\left|\psi_{j}\left(z\right)\right|}^{2}dz,$$

or the total power of the photonic droplet:

(69) $$P=\sum_{n}\int_{-\infty }^{+\infty }{\left|u_{n}\left(x\right)\right|}^{2}dx.$$

For solitons produced by Equations (66) and (67), the sets of control parameters are, respectively, $\left(C,g\right)$ for fixed *N* or $\left(C,P\right)$.

**Figure 20:**
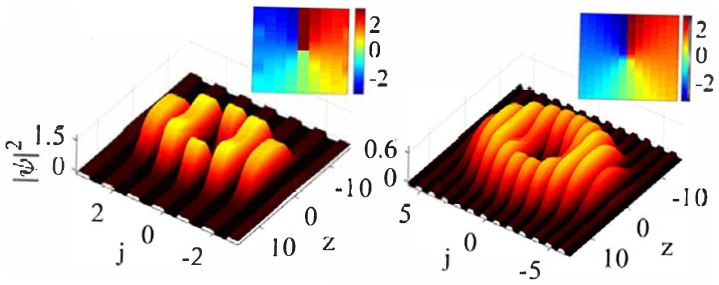
(**Left** and **right**) The panels display the amplitude and phase profiles of stable on-site- and intersite-centered semi-discrete vortex solitons with topological charge S=1, produced by the numerical solution of Equation (66) with parameters $\left(g,C\right)=\left(0.48,0.1\right)$ and $\left(0.77,0.15\right)$, respectively. The norm (68) of both solutions is N=100. The figure is borrowed from Ref. [83].

The models based on Equations (66) and (67) give rise to many families of semi-discrete solitons, including a novel species of semi-discrete vortex solitons. Typical examples of the on-site and intersite vortices with topological charge S=1, produced by Equation (66), are displayed in Figure 20.

**Figure 21:**
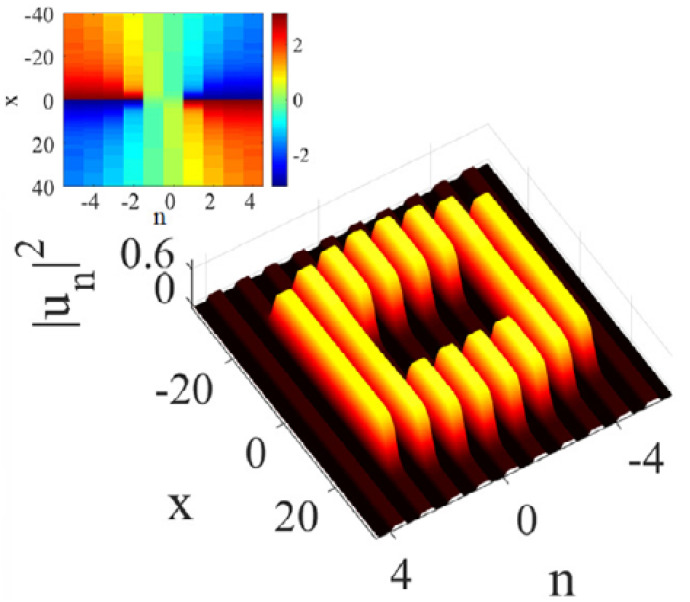
Amplitude and phase profiles of a stable intersite-centered semi-discrete vortex soliton with topological charge S=2, produced by the numerical solution of Equation (67) with C=0.018. The total power (69) of this soliton is P=250. The figure is borrowed from Ref. [83].

An example of a stable semi-discrete vortex soliton produced by Equation (67) with S=2 is displayed in Figure 21.

**Figure 22:**
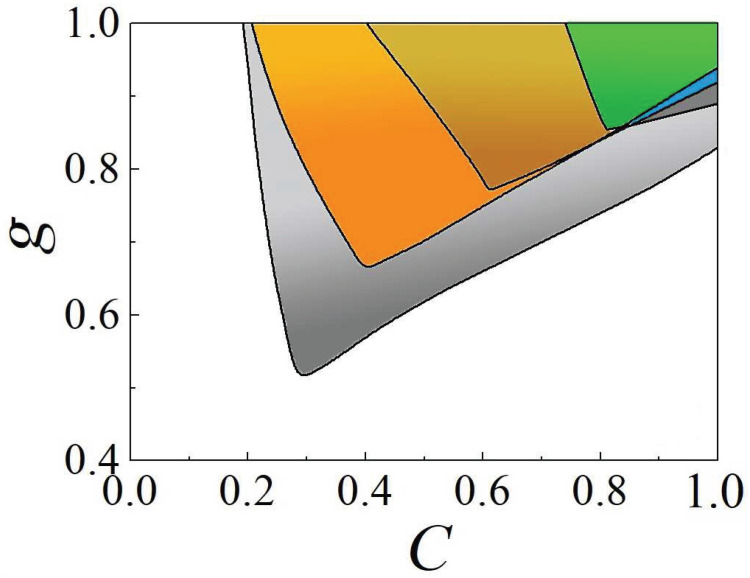
Stability areas in the parameter plane $\left(C,g\right)$, produced by the numerical solution of Equation (66) for on-site-centered semi-discrete vortex solitons with S=2 (all colored regions), 3 (orange + brown + green), 4 (brown + green + blue), and 5 (green + blue + dark gray). For the convenience of plotting, the normalizations for S=2, 3, 4, and 5 are fixed as N=400, 900, 2500, and 4500, respectively. The figure is borrowed from Ref. [83].

Getting back to the semi-discrete system (66), a chart in the plane of $\left(C,g\right)$, which displays the stability areas for the semi-discrete vortex solitons with topological charges S=2, 3, 4, and 5, is plotted in Figure 22. The chart demonstrates abundant multistability; for instance, the stable solitons with S=2 coexist with all higher-order ones (with S=3, 4, and 5).

Self-trapped solutions of a continuum model, which are similar to the semi-discrete vortex solitons outlined above, were recently reported for a photonic crystal built in a $\chi^{\left(2\right)}$ material with a checkerboard structure representing quasi-phase matching [133].

The semi-discreteness of another type is possible in two-component systems, where one component is governed by a discrete equation and the other one by a continuous equation. This type of two-component system was proposed in [134]. It introduced a $\chi^{\left(2\right)}$ model, assuming that the continuous second-harmonic wave propagates in a slab with a continuous transverse coordinate, while the fundamental-harmonic field is trapped in a discrete waveguiding array built on top of the slab.

## 7. Two-Dimensional Fundamental and Vortical Discrete Solitons in a Two-Component PT (Parity-Time) Symmetric Lattice

While the above presentation deals solely with conservative discrete systems, many properties of conservative settings are shared by a very special type of dissipative one, viz. systems with the parity-time (PT) symmetry. They include mutually symmetric spatially separated elements carrying linear gain and loss [135,136,137]. The experimental realization of such systems in optics [137] suggests including the Kerr nonlinearity, thus opening the way to the prediction and creation of PT-symmetric solitons [138,139]. In particular, exact solutions for 1D PT-symmetric solitons and exact results for their stability boundaries were found in the model of the nonlinear PT-symmetric coupler (dual-core waveguide), with mutually symmetric linear gain and loss carried by the linearly coupled cores [140,141]. Stability limits for 2D fundamental solitons in the 2D PT-symmetric coupler with the cubic–quintic nonlinearity in each core (essentially the same as in Equation (67), chosen to prevent the critical collapse instability) were identified in Ref. [142].

The definition of the PT symmetry makes it also natural to consider discrete PT-symmetric systems. Various species of stable discrete solitons were predicted in chains of PT-symmetric elements [89,143,144,145,146,147], and the existence of such solitons was demonstrated experimentally [148].

A natural model for the creation of PT-symmetric discrete 2D solitons is a generalization of the 2D discrete nonlinear coupler, based on Equation (44), by adding the linear gain and loss terms with strength γ>0 to the coupled equations [89]:

(70) $$\begin{array}{ccc} & & i\frac{d\psi_{m,n}}{dz}=-\frac{C}{2}\left(\psi_{m,n+1}+\psi_{m,n-1}+\psi_{m-1,n}+\psi_{m+1,n}-4\psi_{m,n}\right)\\ & & -|\psi_{m,n}{|}^{2}\psi_{m,n}-\varphi_{m,n}+i\gamma \psi_{m,n},\\ & & i\frac{d\varphi_{m,n}}{dz}=-\frac{C}{2}\left(\varphi_{m,n+1}+\varphi_{m,n-1}+\varphi_{m-1,n}+\varphi_{m+1,n}-4\varphi_{m,n}\right)\\ & & -|\varphi_{m,n}{|}^{2}\varphi_{m,n}-\psi_{m,n}-i\gamma \varphi_{m,n}. \end{array}$$

Here, in terms of the optical realization, the evolution variable *z* is the propagation distance, the inter-core coupling coefficient is scaled to be 1, and C>0 is the constant of the intra-core coupling between adjacent sites of the lattice. The dispersion relations for discrete plane-wave solutions to the linearized version of Equations (70), $\left\{\psi_{m,n}\left(z\right),\varphi_{m,n}\left(z\right)\right\}∼\exp\left(ipm+iqn+ikz\right)$, is

(71) $$k=-2C\left(\sin^{2}\frac{p}{2}+\sin^{2}\frac{q}{2}\right)\pm \sqrt{1-\gamma^{2}}.$$

As follows from Equation (71), the PT symmetry holds under the condition $\gamma <\gamma_{\max}\equiv 1$, i.e., the gain–loss strength γ must be smaller than the linear-coupling coefficient, that is 1 in the present notation, which is a generic property of PT-symmetric couplers [140,141].

Stationary modes with the real propagation constant *k* are looked for as solutions to the full nonlinear system of Equations (70) in the usual form, $\left\{\psi_{m,n}\left(z\right),\varphi_{m,n}\left(z\right)\right\}=e^{ikz}\left\{u_{m,n},v_{m,n}\right\}$, with stationary amplitudes obeying the equations:

(72) $$\begin{array}{ccc} \left(k+i\gamma \right)u_{m,n} & = & \frac{C}{2}\left(u_{m,n+1}+u_{m,n-1}+u_{m-1,n}+u_{m+1,n}-4u_{m,n}\right)\\ +|u_{m,n}{|}^{2}u_{m,n}+v_{m,n},\\ \left(k-i\gamma \right)v_{m,n} & = & \frac{C}{2}\left(v_{m,n+1}+v_{m,n-1}+v_{m-1,n}+v_{m+1,n}-4v_{m,n}\right)\\ +|v_{m,n}{|}^{2}v_{m,n}+u_{m,n}. \end{array}$$

Localized states produced by Equation (72) are characterized, as above, by the total power:

(73) $$P=\sum_{m,n}\left(|u_{m,n}{|}^{2}+{\left|v_{m,n}\right|}^{2}\right).$$

**Figure 23:**
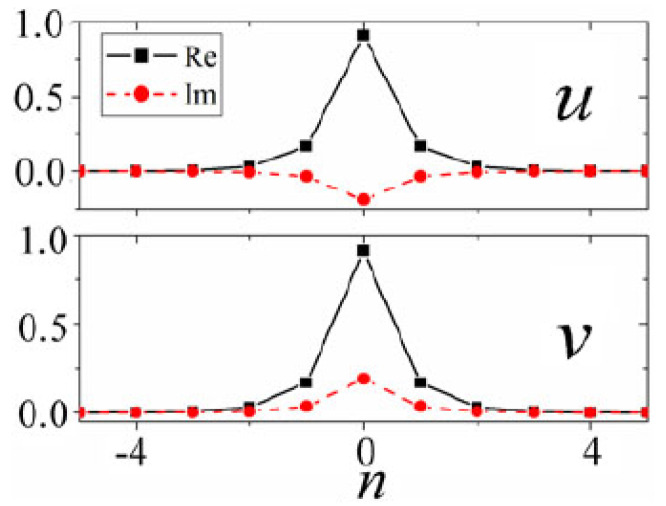
Cross-sections, along m=0, of the real and imaginary parts of components $u_{m,n}$ and $v_{m,n}$ of a stable PT-symmetric fundamental soliton produced by the numerical solution of Equation (75), with (C,γ)=(0.3,0.4), taking the relation (74) into account. The total power (73) of the soliton is P=2. The figure is borrowed from Ref. [89].

Straightforward analysis of Equations (72) demonstrates that the system produces PT-symmetric fundamental-soliton solutions, which must be subject to the relation $v_{m,n}=u_{m,n}^{*}$ (with * standing for the complex conjugate), in the form of

(74) $$\left\{u_{m,n},v_{mn}\right\}=w_{m,n}\exp\left(\pm (i/2)\arcsin\gamma \right),$$

where the real discrete distribution $w_{m,n}$ should be found as a solution of the usual stationary equation for 2D discrete solitons:

(75) $$\left(-k+\sqrt{1-\gamma^{2}}\right)w_{m,n}+\frac{C}{2}\left(w_{m,n+1}+w_{m,n-1}+w_{m-1,n}+w_{m+1,n}-4w_{m,n}\right)+w_{m,n}^{3}=0;$$

cf. Equation (38). In agreement with the linear spectrum (71), Equation (75) may produce soliton solutions for $k>\sqrt{1-\gamma^{2}}$. An example of a stable fundamental PT-symmetric soliton is displayed, by means of its cross-section shapes, in Figure 23.

**Figure 24:**
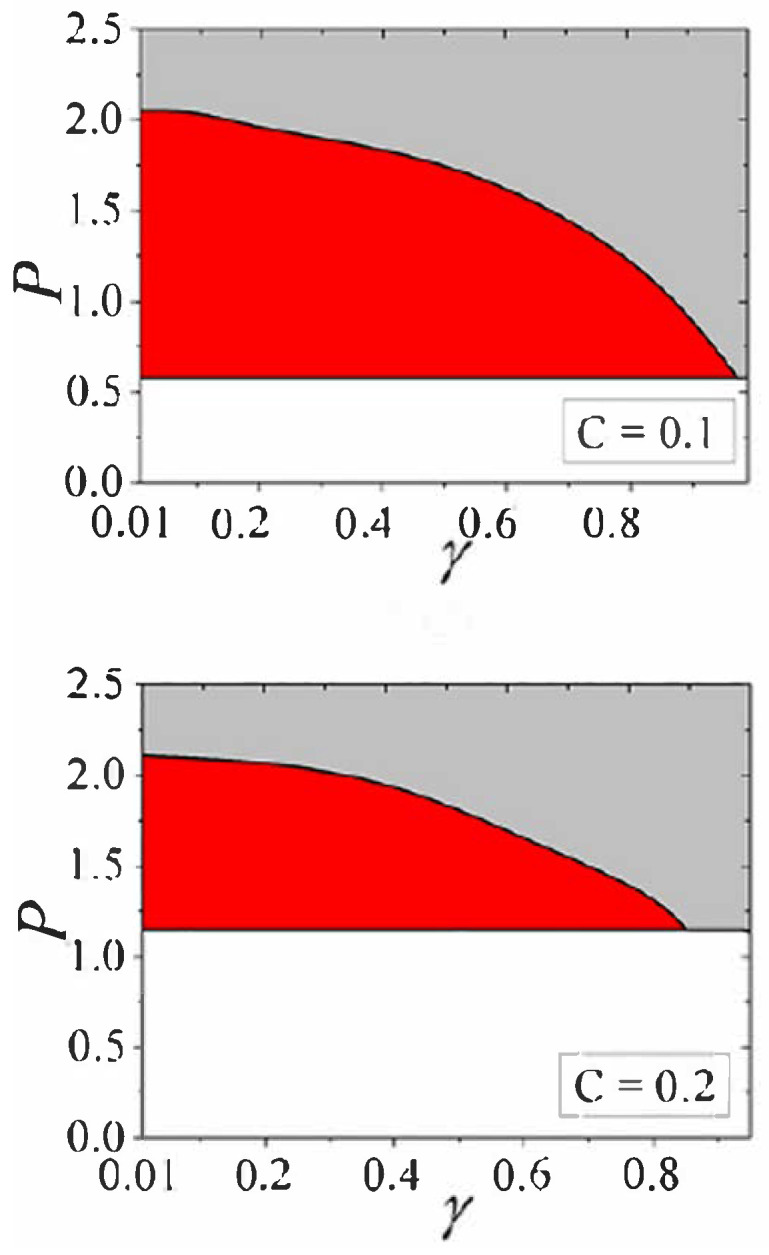
Red and gray colors designate, respectively, stability and instability areas for PT-symmetric fundamental discrete solitons, produced by Equations (74) and (75), in the plane of the gain–loss coefficient, γ, and total power *P*, which is defined as per Equation (73). The soliton solutions do not exist in the white area. The figure is borrowed from Ref. [89].

The existence and stability of the PT-symmetric fundamental discrete solitons is summarized in the plane of $\left(\gamma ,P\right)$ for the fundamental solitons in Figure 24. It is seen that, naturally, the stability area shrinks as the gain–loss coefficient γ is approaching its limit value, $\gamma_{\max}=1$ (cf. the 1D situation considered in Refs. [140,141]). The existence boundary, i.e., the minimum value of *P*, below which no solitons are found (in the white area), corresponds to the limit of very broad small-amplitude solitons. In this limit, the discrete soliton may be approximated by its counterpart in the continuum NLS equation, i.e., the above-mentioned Townes soliton, whose power takes the unique value, which, thus, determines the existence boundary in Figure 24.

The stability boundary in Figure 24 may be understood as the one at which the symmetric soliton is destabilized by the spontaneous symmetry breaking (as described in detail above for 2D solitons produced by the linearly coupled conservative DNLS equations (44); see also Ref. [20]), which is modified here by the presence of the linear gain and loss. Because asymmetric solitons cannot exist in the system with the balanced gain and loss, the symmetry breaking always leads to either the blowup or decay of the soliton [89]. In their stability region, the PT-symmetric fundamental discrete solitons actually represent the system’s ground state [89].

**Figure 25:**
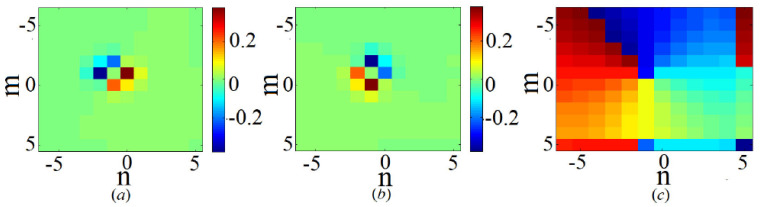
Real (**a**) and imaginary (**b**) parts, and the phase structure (**c**), of the field $u_{m,n}$ of a stable discrete PT-symmetric vortex soliton, produced by Equation (72) for (C,γ)=(0.06,0.4), with propagation constant k=1 and total power P=1.65, defined as per Equation (73). The figure is borrowed from Ref. [89].

Alongside the fundamental discrete PT-symmetric solitons, the same system of equations (72) produces PT-symmetric vortex solitons, which also have their stability area; see the details in Ref. [89]. An example of a stable PT-symmetric vortex soliton is presented in Figure 25.

In addition to the PT-symmetric solitons, Equation (72) gives rise to anti-PT-symmetric ones, defined by relation $v_{m,n}=-u_{m,n}^{*}$. They, as well as anti-PT-symmetric vortex solitons, are stable in some parameter areas (see the details in Ref. [89]), but those areas are essentially smaller than their counterparts for the PT-symmetric modes. The reduced stability area for the anti-PT-symmetric fundamental solitons is explained by the fact that they cannot be the system’s ground state.

## 8. Conclusions

The interplay of the discreteness and intrinsic nonlinearity in various physical media—chiefly, in nonlinear optics and BECs—gives rise to a great variety of self-trapped localized states, in the form of discrete solitons. This article aims to produce a concise review, starting from the brief survey of basic theoretical models combining the discreteness in 1D, 2D, and 3D geometries and various nonlinearities, such as cubic, quadratic, and quintic. The main subject addressed in the article is a summary of the basic results for 2D and 3D discrete solitons produced by such models. Unlike the topic of 1D discrete solitons, the multidimensional ones were not previously reviewed in a systematic form. Along with the fundamental solitons, topologically organized ones, in the form of solitary vortices and discrete skyrmions, are considered as well. Some experimental findings are also included, such as the observation of 2D discrete optical solitons with embedded vorticity.

In many cases, the discreteness helps to produce states that either do not exist or are definitely unstable in continuum analogs of the discrete settings. In particular, these are 2D fundamental and vortex solitons, which may be stable in the discrete form, while their continuum counterparts are completely unstable in the free space. On the other hand, the mobility of solitons, which is their obvious property in the continuum, is a nontrivial issue for the lattice (discrete) solitons.

The work in this area remains a subject of ongoing theoretical and experimental work, promising new findings. A prospective direction is to produce 2D and 3D self-trapped states with intrinsic topological structures. Some results obtained in this direction are presented in this article, such as discrete solitons in the system with spin–orbit coupling [120] (see also Ref. [149]), sophisticated 3D discrete modes with embedded vorticity [122,123], and discrete skyrmions [127]. A challenging task is the experimental realization of these states, which, thus far, were only predicted in the theoretical form.

It is relevant to mention some topics that may be relevant in the present context, but are not included here, to keep a reasonable size of the review. In particular, these are interactions of discrete solitons with local defects in the underlying lattice, as well as with interfaces and edges. It is known that defects and surfaces may often help to create and stabilize localized modes that do not exist or are unstable in uniform lattices, such as Tamm [150] and topological-insulator [151,152] states. Not included either are 2D discrete models with the fractional coupling between lattice sites [153].

Another vast area of studies, which is not considered here, deals with dissipative discrete nonlinear systems. In this article, only the very special case of PT-symmetric systems is addressed. Basic nonlinear dissipative models are represented by discrete complex Ginzburg–Landau equations, i.e., DNLS equations with complex coefficients in front of the on-site linear and nonlinear terms, which account for losses and compensating gain [154]. Unlike conservative and PT-symmetric models, the dissipative ones may only give rise to stable discrete solitons, which do not exist in continuous families, but rather as isolated *attractors* [155,156,157].
